# Choice-correlated activity fluctuations underlie learning of neuronal category representation

**DOI:** 10.1038/ncomms7454

**Published:** 2015-03-11

**Authors:** Tatiana A. Engel, Warasinee Chaisangmongkon, David J. Freedman, Xiao-Jing Wang

**Affiliations:** 1 Department of Neurobiology, Yale University School of Medicine, Kavli Institute for Neuroscience, 333 Cedar Street, New Haven, Connecticut 06510, USA; 2 Department of Bioengineering, Stanford University, 318 Campus Drive, Stanford, California 94305, USA; 3 Department of Neurobiology, The University of Chicago, 5812 S. Ellis Ave., Chicago, Illinois 60637, USA; 4 Center for Neural Science, New York University, 4 Washington Place, New York, New York 10003, USA; 5 NYU-ECNU Joint Institute of Brain and Cognitive Science, NYU-Shanghai, Shanghai 200122, China

## Abstract

The ability to categorize stimuli into discrete behaviourally relevant groups is an essential cognitive function. To elucidate the neural mechanisms underlying categorization, we constructed a cortical circuit model that is capable of learning a motion categorization task through reward-dependent plasticity. Here we show that stable category representations develop in neurons intermediate to sensory and decision layers if they exhibit choice-correlated activity fluctuations (choice probability). In the model, choice probability and task-specific interneuronal correlations emerge from plasticity of top-down projections from decision neurons. Specific model predictions are confirmed by analysis of single-neuron activity from the monkey parietal cortex, which reveals a mixture of directional and categorical tuning, and a positive correlation between category selectivity and choice probability. Beyond demonstrating a circuit mechanism for categorization, the present work suggests a key role of plastic top-down feedback in simultaneously shaping both neural tuning and correlated neural variability.

## Abstract (web summary)

The ability to categorize stimuli into discrete behaviourally relevant groups is an essential cognitive function. Here, the authors demonstrate a critical role for choice-correlated activity fluctuations in the emergence of stable cortical category representations.

## Graphical abstract

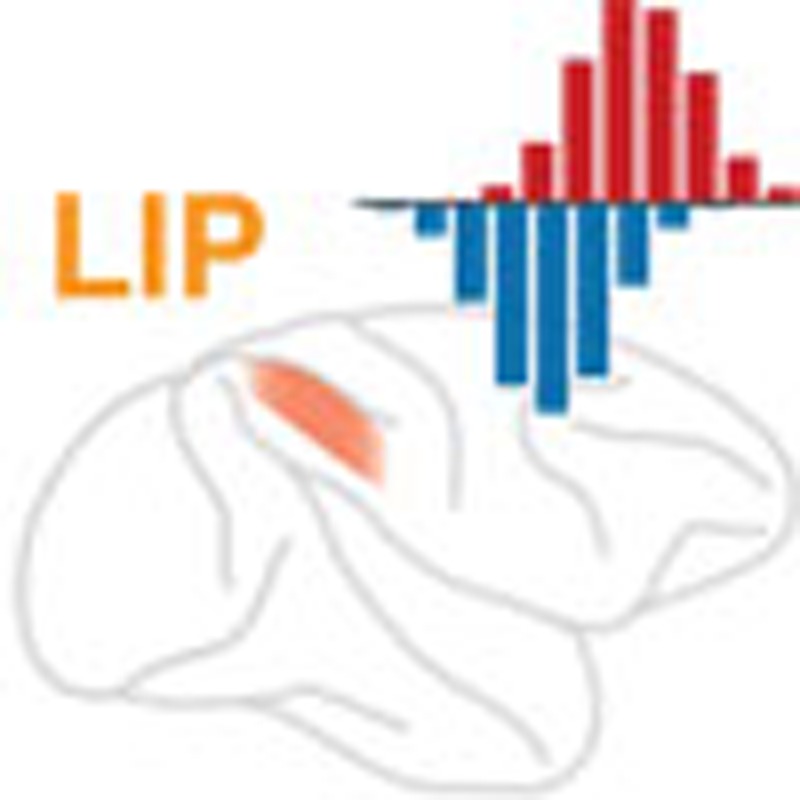

Through experience we can learn to classify a continuum of sensory stimuli into discrete meaningful categories, which are critical for guiding behaviour^1^,^2^. Training improves our ability to discriminate stimuli belonging to different categories and to group together perceptually dissimilar items within the same category. Such learning and refinement of categorical discriminations occur continuously in everyday life; however, their neural basis is poorly understood.

During training on visual tasks, perceptual improvements are accompanied by only moderate tuning changes in the early visual cortex^3^,^4^, whereas more dramatic changes occur in inferior temporal and posterior parietal cortices. In monkeys trained to classify directions of random dot motion into two arbitrary categories, neurons in the lateral intraparietal (LIP) area encoded learned motion categories in an almost binary manner^5^, whereas in naive animals LIP neurons represent directions uniformly with bell-shaped tuning functions^6^. In contrast, categorization training did not induce any apparent change in motion tuning of neurons in the middle temporal (MT) area. Similarly, changes in responses of LIP but not MT neurons were associated with improved behavioural sensitivity on visual discrimination tasks^7^,^8^,^9^, which had been attributed to refinements of functional connectivity between MT and LIP through reinforcement learning^10^,^11^; however, the underlying circuit mechanism remains unknown.

We examined whether changes in tuning of LIP neurons induced by training on a motion categorization task can emerge in a neural circuit model through biophysically plausible Hebbian synaptic plasticity modulated by reward prediction error (RPE) signals^12^,^13^,^14^,^15^. Unlike the classical two-layer categorization model^16^, our model incorporated a layer of neurons intermediate to sensory and decision layers. We found that neurons in the intermediate layer develop stable category representation if fluctuations of their firing rates are correlated with behavioural choices. In contrast, behavioural performance and neuronal tuning deteriorate with training in networks where activity fluctuations are not correlated with choices. Weak but systematic correlations between neural fluctuations and choices, termed choice probability (CP), have been found in many cortical areas^17^,^18^. Here we show that CP is critical for successful learning through reward-dependent Hebbian plasticity, which generally holds across different network architectures and behavioural tasks.

Our model predicts that a mixture of directional and categorical tuning and bimodal distribution of preferred directions emerge in the intermediate-layer neurons through learning. This prediction was confirmed by analysis of LIP responses recorded in monkeys trained on the motion categorization task. Moreover, the model predicts that neurons with larger CP exhibit a larger increase in their category sensitivity (CS), leading to a positive correlation between these measures, which was also found in the LIP data. Finally, the model suggests that task-specific noise correlations arise from the plasticity of top-down connections and makes testable predictions about changes of noise correlations throughout learning.

## Results

### A neural circuit model of category learning

**Figure 1:**
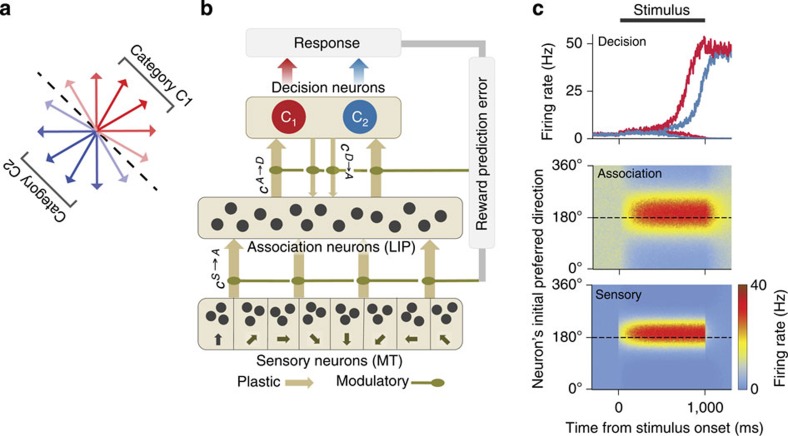
Categorization task and the neural circuit model. (**a**) A set of 12 motion direction stimuli is divided into two categories, C1 and C2 (red and blue arrows), separated by a category boundary (black dashed line). On each trial, one randomly chosen motion stimulus is presented, and the model learned through trial and error to indicate its category membership. (**b**) Schematic of the circuit model. The network comprises a sensory (MT), an association (LIP) and a decision neural circuits. Neurons in the sensory circuit are tuned to motion directions (indicated by arrows). They receive directional bottom-up inputs and provide inputs to the association neurons through feedforward synapses (*c^S→A^*). The decision circuit (C_1_ and C_2_ populations) pools activity of association neurons through feedforward synapses (*c^A→D^*) and generates a category decision through competitive attractor dynamics. The model has feedback connections from the decision to association neurons (*c^D→A^*). All synaptic connections between the local circuits undergo Hebbian plasticity modulated by a reward prediction error signal. (**c**) An example network activity before categorization training. A motion direction stimulus (195°) is presented for 1 s (grey bar). The sensory and association neurons show direction-tuned responses in their spatiotemporal activity patterns (lower and middle panels, respectively). *x* axis, time; *y* axis, neurons labelled by the preferred direction; firing rate is colour-coded. The decision circuit generates categorical choice through a winner-take-all competition between the C_1_ and C_2_ populations (upper panel). Firing rates of the C_1_ and C_2_ populations are shown for two trials, where *C*_1_ (red line) and *C*_2_ (blue line) choice was made for the same stimulus.

We trained a neural circuit model to perform a motion categorization task^5^. Twelve motion directions were assigned to two categories, C1 and C2, defined by an arbitrary category boundary (Fig. 1a), and the model learned through trial and error to decide on the category membership of these stimuli.

Our model is a recurrent neural network comprising three interconnected circuits (Fig. 1b). Sensory neurons (MT) encode motion directions with bell-shaped tuning functions (Fig. 1c), arising from direction-selective bottom-up inputs and structured recurrent excitation^19^. Association neurons (LIP) are also tuned to motion directions initially (Fig. 1c)—just like LIP neurons in naive monkeys^6^—because synaptic weights are initialized to be stronger between sensory and association neurons with similar preferred directions. Over the course of learning, tuning of association neurons changes through synaptic plasticity. The activity of association neurons is pooled by the decision network, which consists of two competing populations (C_1_ and C_2_, Fig. 1b,c) firing at higher rates for the two respective category decisions^20^,^21^. These neurons encode the model’s choice and represent a subpopulation of neurons within LIP or in the prefrontal cortex. Synaptic connections between association and decision neurons are initialized at random values; therefore, the model’s categorization decisions are completely random initially.

Our model has plastic feedforward connections from sensory to association (*c^S→A^*) and from association to decision (*c^A→D^*) circuits, and plastic feedback connections from decision to association circuit (*c^D→A^*, Fig. 1b). At the end of each trial, the strength *c* of each plastic synapse is updated according to a reward-dependent Hebbian plasticity rule:


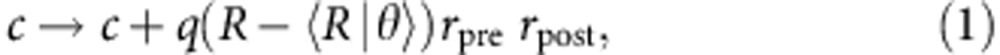


where *r*_pre_ and *r*_post_ are the trial-average firing rates of pre- and postsynaptic neurons, *q* is the learning rate parameter, *R* is the reward received on each trial (1 or 0 for correct and incorrect decisions, respectively), *θ* stands for a motion direction stimulus and ‹*R*|*θ*› is a stimulus-specific reward expectation, which may be encoded in the orbitofrontal cortex or basal ganglia. For simplicity, we computed ‹*R*|*θ*› as a running average of reward history^14^. Phasic activity of dopamine neurons encodes the difference *R*−‹*R*|*θ*›, called the RPE signal^12^,^22^,^23^, and dopamine concentration modulates long-term plasticity^24^,^25^. In our model, positive RPE signals lead to potentiation, while negative RPE signals lead to depression. Finally, the synaptic strengths *c* are bounded between 0 and 1.

### Model learning performance

**Figure 2:**
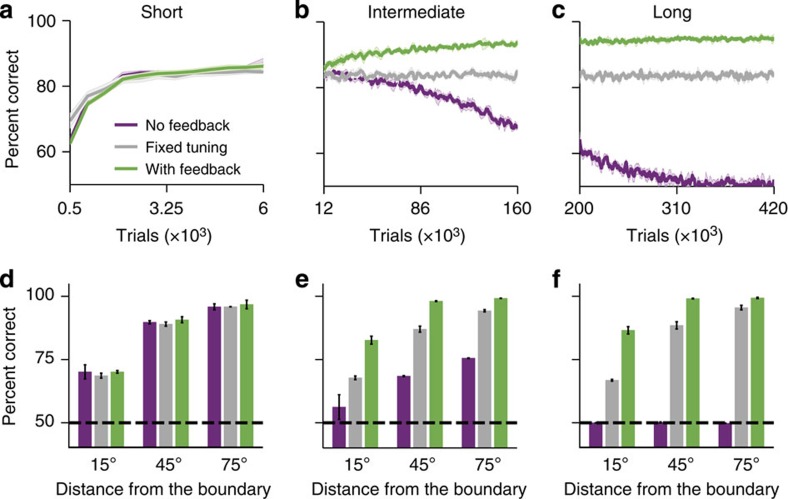
Behavioural performance of the network models during training on the motion categorization task. Performance of the networks with feedback (green), without feedback (purple) and with fixed tuning of association neurons (grey) is shown at three stages of learning: short (**a**,**d**), intermediate (**b**,**e**) and long (**c**,**f**). (**a**–**c**) Overall percent correct responses as a function of the number of trials performed. (**d**–**f**) Psychometric functions evaluated at the end of each training epoch: percent correct responses for stimuli close to (15°) and farther from (45° and 75°) category boundary. At the short training stage, performance improved equally in all three models. As the training progressed, performance of the network with feedback steadily improved especially for the near-boundary stimuli, while performance of the network without feedback gradually deteriorated and eventually dropped to the chance level. Performance of the network with fixed tuning of association neurons remained at the level attained by the end of the short training stage. Shaded area in **a**–**c** and error bars in **d**–**f** indicate s.d. across five independent realizations of each network type.

We compared the learning performance of our model with that of two control networks: a network without feedback, which had only feedforward connections between the local circuits, and a network with fixed tuning of association neurons, which had only feedforward connections and no plasticity of synapses between sensory and association neurons (effectively, a classical two-layer categorization model^16^). Initially, performance of all models rapidly improved from the chance level to ~80% correct responses over several thousand trials (Fig. 2a). During this short period of associative learning, the models learn to associate motion directions and categories, driven by plasticity of the synapses from association to decision neurons. Plasticity transforms the profile of these synapses from random to nearly binary: association neurons with preferred directions in category C1 have strong weights to C_1_ and nearly zero weights to C_2_ decision neurons, and *vice versa* (Supplementary Fig. 1b). As a result, motion directions from category C1 generate stronger input into the C_1_ decision population, which makes *C*_1_ choices more likely, because the probability of choice in our model is determined by the difference in input currents to two competing populations^21^. At this stage of learning, the performance is less accurate for stimuli closest to (15°) the category boundary (Fig. 2d). Near-boundary stimuli activate a subpopulation of association neurons with preferred directions in both categories (Fig. 1c), resulting in comparable inputs to both decision populations and less reliable categorization behaviour.

As training progressed, the three models began to exhibit markedly different performance trends (Fig. 2b,e). The network with feedback steadily improved performance over a hundred thousand trials (several months of training for monkeys), mainly due to increasing accuracy for the near-boundary stimuli (Fig. 2e). In contrast, the performance of network without feedback gradually deteriorated, whereby accuracy decreased for all motion directions. The network with fixed tuning of association neurons maintained the same performance level as attained by the end of the associative learning period. These performance trends were preserved throughout extensively long training (Fig. 2c,f), by the end of which the performance of the network without feedback dropped to the chance level.

### Transformation of tuning in association neurons

**Figure 3:**
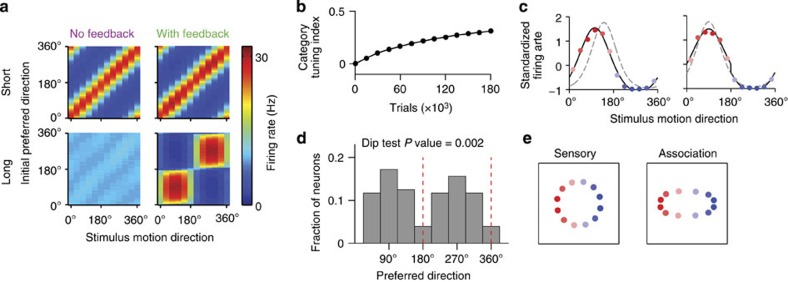
Mixed direction and category tuning emerges in association neurons through learning. (**a**) Tuning profiles of association neurons after short (6 × 10^3^ trials, upper row) and long (420 × 10^3^ trials, lower row) periods of training for the network without feedback (left column) and with feedback (right column). *x* axis, stimulus motion direction; *y* axis, neurons arranged and labelled by their preferred direction before learning; firing rate is colour-coded. After extensive categorization training, motion tuning deteriorates in the network without feedback, whereas categorical tuning develops in the network with feedback. (**b**) The average category-tuning index of association neurons steadily increases over the course of training. (**c**–**e**) Mixed direction and category tuning of the association neurons at the intermediate stage of training (65 × 10^3^ trials). (**c**) Tuning profiles of two example association neurons before (grey dashed line) and after learning (coloured dots—firing rates, black solid line—best-fitted tuning function). Tuning curves broaden (right panel) and shift towards category centres (left panel) in neurons with initial preferred directions near category centres and category boundaries, respectively. (**d**) Bimodal distribution of preferred directions in direction-tuned association neurons (Hartigan’s dip test, *P*=0.002). Majority of neurons are tuned away from the category boundary (indicated by red dashed lines). (**e**) Multidimensional scaling analysis reveals a circular configuration of motion directions in the representation of sensory neurons (left panel), and an elliptical configuration elongated along the axis perpendicular to the category boundary in the representation of association neurons (right panel).

The striking differences in learning performance of the three models cannot be explained by the synaptic connections from the association to decision neurons, as they are shaped equally in all networks during associative learning and remain virtually unchanged later on (Supplementary Fig. 1b). The reason for the observed performance differences is the change in tuning of association neurons, driven by the plasticity of synapses between sensory and association neurons (Supplementary Figs 1 and 2). In the networks with and without feedback, association neurons have initially the same uniform direction tuning, which is only slightly altered after a short period of learning (6,000 trials, Fig. 3a, upper row), but becomes dramatically different in the two models after extensively long training (420,000 trials, Fig. 3a, lower row). In the network without feedback, the direction tuning deteriorates: the association neurons fire at the same rate for all motion directions. Consequently, the decision circuit receives nonselective inputs and the performance is at the chance level. In contrast, tuning transforms from directional to categorical in the network with feedback: two nonoverlapping subpopulations emerge in the association circuit that respond selectively to stimuli from their preferred categories. As a result, category decisions are very accurate even for near-boundary stimuli.

To quantify the development of category selectivity throughout learning, we computed the average category-tuning index^5^ (CTI) of association neurons in the model with feedback. Categorical tuning entails that neurons respond differently to stimuli in different categories and do not differentiate between stimuli in the same category. Accordingly, the CTI varies from −1.0 to 1.0, where positive values indicate larger response differences for stimuli in different categories and negative values indicate larger differences within each category (see Methods). Before learning, the average CTI of association neurons was zero, indicating uniform direction tuning (Fig. 3b), and then CTI gradually increased. At the intermediate learning stage corresponding to the amount of categorization training received by monkeys (65,000 trials or ~10–12 weeks), the average CTI was 0.18, comparable to the CTI value 0.125 previously reported for LIP neurons^5^.

The gradual increase in the CTI was accompanied by changes in the tuning curves of individual association neurons, which followed two systematic trends. In neurons that initially preferred directions near category centres, tuning curves broadened (Fig. 3c, right), while in neurons that initially preferred directions near category boundaries, tuning curves shifted so that their preferred directions moved towards centres of the respective categories (Fig. 3c, left). Broadening and shifting of tuning curves led to mixed tuning, whereby direction and category signals were combined on the single-cell level. To quantify this mixture, we fitted the tuning curve of each association neuron with a generalized linear model (GLM)^26^, which contained a linear combination of two regressor functions: a direction (bell-shaped, equation (12)) and a category (binary step-like, equation (13)) tuning profiles (see Methods). The tuning was classified as pure directional, pure categorical or mixed, according to GLM coefficients that were significantly different from 0. At the intermediate learning stage (65,000 trials), 15.6% of association neurons exhibited a significant influence of category on their tuning curves, while 84.4% remained purely direction-tuned. We examined the distribution of preferred directions in direction-tuned neurons, and found that more neurons were tuned to category centres than to category boundaries (Fig. 3d, the result did not change if all neurons were included).

Broadening and shifting of tuning curves alter the representation of motion directions in a way that facilitates the discrimination of categories. We visualized the ensuing representation on the population level using classical multidimensional scaling^27^ (MDS). In this framework, stimuli are represented as vectors in a high-dimensional space of neural firing rates, where each dimension corresponds to a neuron in the population. The MDS algorithm finds a two-dimensional configuration of the stimuli that preserves the distances between them as much as possible. In the sensory circuit, the MDS algorithm yields a circular configuration (Fig. 3e, left) that faithfully reproduces the arrangement of directions in the physical space. In the association circuit, the configuration is elongated along the axis perpendicular to the category boundary (Fig. 3e, right), which increases the distances between near-boundary stimuli in different categories making them more easily discriminable and decreases distances between stimuli within the same category making them less discriminable.

### Mixed direction and category tuning in LIP neurons

We compared tuning changes in our model to the tuning (during the period of stimulus presentation) of MT and LIP neurons recorded in monkeys trained to categorize motion directions^5^. Such a comparison is meaningful, if the model and monkeys experienced similar amount of categorization training and reached similar behavioural performance. In the model, the time course of learning depends on the learning rate *q* and the maximal strength of feedback connections 
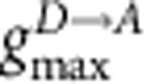
 (Supplementary Fig. 3). We simulated the model for a range of *q* and 
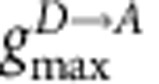
 and used the parameters that provided good match to experimental data for the similar number of training trials (that is, 65,000 trials, see Supplementary Fig. 4).

**Figure 4:**
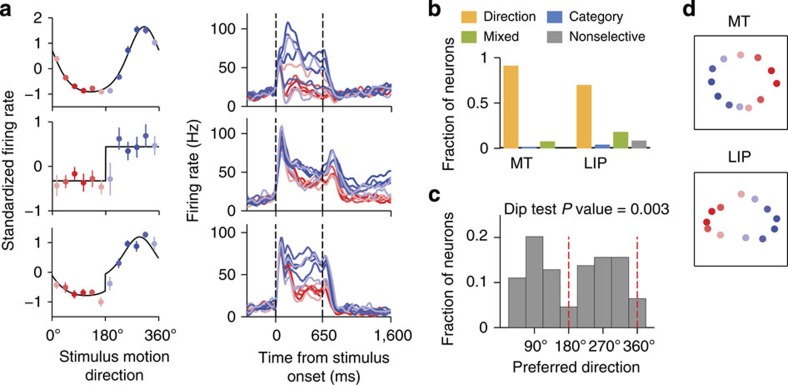
Mixed direction and category tuning in LIP neurons. (**a**) Average response to the 12 motion directions for three example LIP neurons, classified as direction-tuned (upper row), category-tuned (middle row) and mixed direction and category-tuned (lower row). Left column: tuning curves, the average standardized firing rates during stimulus presentation (coloured dots) overlaid by the best-fitted tuning function (black line). Error bars indicate s.e.m. Right column: average firing rate traces in response to stimuli from category C1 (red lines) and C2 (blue lines). (**b**) The fraction of MT (*N*=67) and LIP (*N*=156) neurons that exhibited directional tuning, category tuning, mixed tuning or were not stimulus-selective. (**c**) Bimodal distribution of preferred directions in direction-tuned LIP neurons (Hartigan’s dip test, *P*=0.003, *N*=109), in support of the model prediction (Fig. 3d). (**d**) Multidimensional scaling analysis reveals a nearly uniform representation of motion directions in the MT population (upper panel), and an elongated representation in the LIP population (lower panel), where the distances are larger between the stimuli from different categories, *c.f*. the model prediction in Fig. 3e.

We fitted the tuning curve of each neuron in our database (67 MT and 156 LIP neurons) with direction and category-tuning functions and then classified tuning as directional, categorical or mixed following the same procedure that was used for model neurons. The majority of MT (91.0%) and LIP neurons (69.9%) exhibited pure direction tuning (Fig. 4a, upper panels, Fig. 4b). In agreement with our model prediction, the distribution of preferred directions was significantly bimodal among direction-tuned LIP neurons (Hartigan’s dip test *P*=0.003, Fig. 4c), but not among MT neurons (Hartigan’s dip test *P*=0.08). A considerable fraction of LIP neurons (18.0%) showed a mixture of directional and categorical tuning (Fig. 4a, lower panels). The distribution of preferred directions remained significantly bimodal when the mixed-tuned LIP neurons were included in the analysis (Hartigan’s dip test *P*<10^−7^). A small fraction of LIP neurons (3.9%) exhibited pure category tuning (Fig. 4a, middle panels), and the rest (8.3%) were not stimulus-selective. As a control, we repeated the analyses in different time epochs during the trial (Supplementary Table 1) and using a smoothed category-tuning function (Supplementary Table 2), and obtained similar results.

The representation of motion directions at the population level was consistent with the model prediction as well: the MDS algorithm revealed a nearly circular configuration of motion directions in MT (Fig. 4d, upper panel), whereas in LIP motion directions were arranged on an elongated ellipse with the major axis perpendicular to the category boundary (Fig. 4d, lower panel, see Supplementary Note 1 for statistical significance test). Similarly, CTI was significantly higher in LIP than in MT as has been previously reported for the same dataset^5^. Although the LIP population demonstrated high heterogeneity, the main tuning features in LIP bear a remarkable resemblance to the tuning transformation induced by learning in our model.

### Reward-driven learning depends on choice probability

To understand effects of learning on tuning of association neurons, we need to examine the reward-dependent Hebbian plasticity rule (equation (1)). The plasticity rule entails that the expected weight change for each stimulus ‹Δ*c*|*θ*› is proportional to the covariance between the reward *R* and neural activity *N*=*r*_pre_ *r*_post_ (ref. 15) (see Supplementary Note 2):


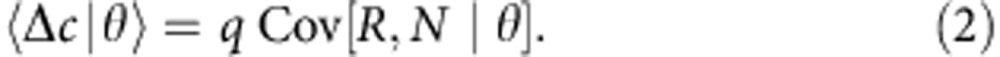


This means that average synaptic weight changes across many trials are driven by covariation between trial-to-trial fluctuations of the firing rates and reward. Thereby, synapses change to increase the expected reward. If for a particular synapse the neural activity is systematically higher on trials when the reward is above its mean, then the covariance is positive, the synapse is potentiated, and hence the mean neural activity and the expected reward increase (and analogously for negative covariance). Fluctuations of both reward and neural activity are critical for learning: if either *R* or *N* is deterministic, the covariance equals zero and learning does not increase expected reward.

Covariation between neural activity and reward entails covariation between neural activity and choices, if reward is assigned on the basis of behavioural responses. This simple intuition can be formalized mathematically, if we express the covariance Cov[*R*,*N*|*θ*] in terms of expectations conditioned on choices. For tasks with only two possible choices, we obtain a simple expression (see Methods for derivation and generalization to arbitrary number of choices):


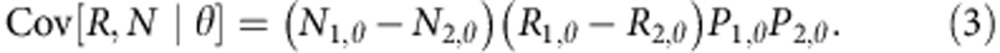


Here *P*_*i*,*θ*_ is the probability that *C*_*i*_ choice is made for the stimulus *θ*; *R*_*i*,*θ*_=‹*R*|*θ*,*C*_*i*_› is the reward expected for choosing *C*_*i*_ for stimulus *θ*; and *N*_*i*,*θ*_=‹*N*|*θ*,*C*_*i*_› is the expected neural activity conditioned on the stimulus *θ* and choice *C*_*i*_.

**Figure 5:**
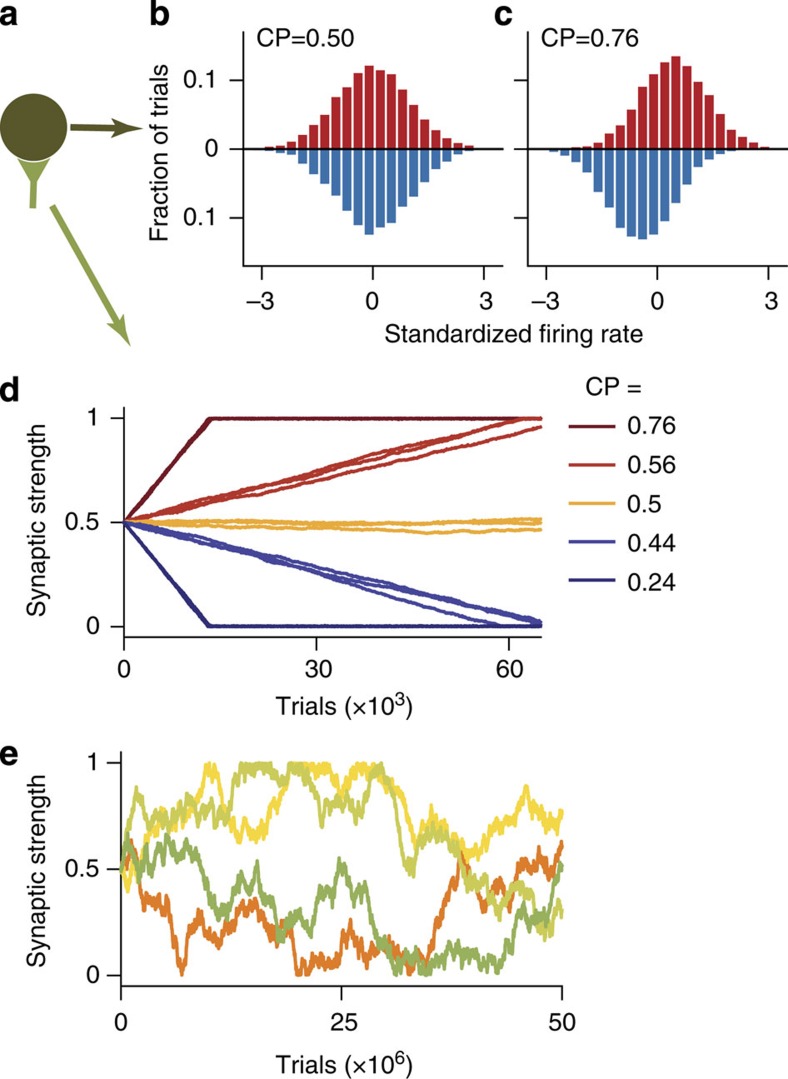
Choice probability determines the direction and magnitude of synaptic changes. (**a**) Schematic of a toy model: firing rate of a neuron is correlated with choices in a behavioural task, where choice *C*_1_ is rewarded and *C*_2_ is not. Synapse of this neuron is updated according to reward-dependent Hebbian plasticity. (**b**,**c**) Firing rate distributions of a toy-model neuron on trials when different choices are made (*C*_1_—red bars, *C*_2_—blue bars). CP is close to 0.5, if the distributions largely overlap, that is, rate fluctuations are not correlated with choices (**b**). CP deviates from 0.5 towards 1 (or 0) when the distributions are well separated, that is, rate fluctuations are correlated with choices (**c**). (**d**) The sign of CP−0.5 determines the direction of synaptic changes: CP>0.5 leads to potentiation and CP<0.5 leads to depression (CP is computed relative to the rewarded choice *C*_1_). The larger is the deviation of CP from 0.5, the faster is the rate of synaptic changes. Synaptic strengths are shown across learning trials for different CP values over an intermediate period of learning (three independent realizations for each CP value). (**e**) Synaptic strengths are shown across many learning trials for CP≈0.5 (corresponds to yellow traces in **d**, note the difference in scale of the *x* axis between **d** and **e**). Synaptic changes are random, and any weight becomes equally likely over a long period of learning. Four independent realizations are shown.

The term (*N*_1,*θ*_−*N*_2,*θ*_) represents the difference between the means of two neural activity distributions obtained on trials when different choices are made for the same stimulus *θ*, and is monotonically related to a measure called choice probability^17^,^28^ (CP, Supplementary Fig. 5a). CP quantifies the accuracy with which an ideal observer could predict choices given neuronal firing rates on a trial-by-trial basis. A CP of 0.5 indicates no correlation between neural fluctuations and choices (*N*_1,*θ*_≈*N*_2,*θ*_, Fig. 5b), whereas a CP of 1 (or 0) indicates that the neuron’s firing rate is always higher (or lower) on trials when *C*_1_ is chosen than on trials when *C*_2_ is chosen for the same stimulus *θ* (*N*_1,*θ*_>*N*_2,*θ*_ in Fig. 5c; our convention of computing CP differs from refs 17, 29, see Supplementary Note 3).

Equation (3) demonstrates that synaptic updates lead to increase in expected reward if CP≠0.5 for pre- or postsynaptic neurons; however, if CP≈0.5 for both pre- and postsynaptic neurons, the covariance Cov[*R*,*N*|*θ*] vanishes irrespective of the reward expectation. This result is a general property of reward-modulated Hebbian plasticity and holds across different tasks and network architectures. It can be illustrated using a single toy-model neuron (Fig. 5a), whose firing rates for *C*_1_ and *C*_2_ choices are sampled from two Gaussian distributions with different means, without specifying mechanisms generating CP. We assumed that *C*_1_ choices are rewarded, leaving other task details unspecified. The synapse of this toy-model neuron is updated according to the reward-modulated Hebbian plasticity rule. As predicted by equation (3), CP determines the direction and magnitude of synaptic changes in the toy model. If CP>0.5, the covariance Cov[*R*,*N*] is positive and the synapse is potentiated (red traces in Fig. 5d), and if CP<0.5 the synapse is depressed (blue traces in Fig. 5d). The covariance magnitude is larger for larger |CP−0.5|, resulting in faster synaptic changes. If CP≈0.5, the covariance vanishes; hence, synaptic modifications are driven by noise similar to a random walk (yellow traces in Fig. 5d) and over a long period of learning any synaptic weight becomes equally likely (Fig. 5e).

**Figure 6:**
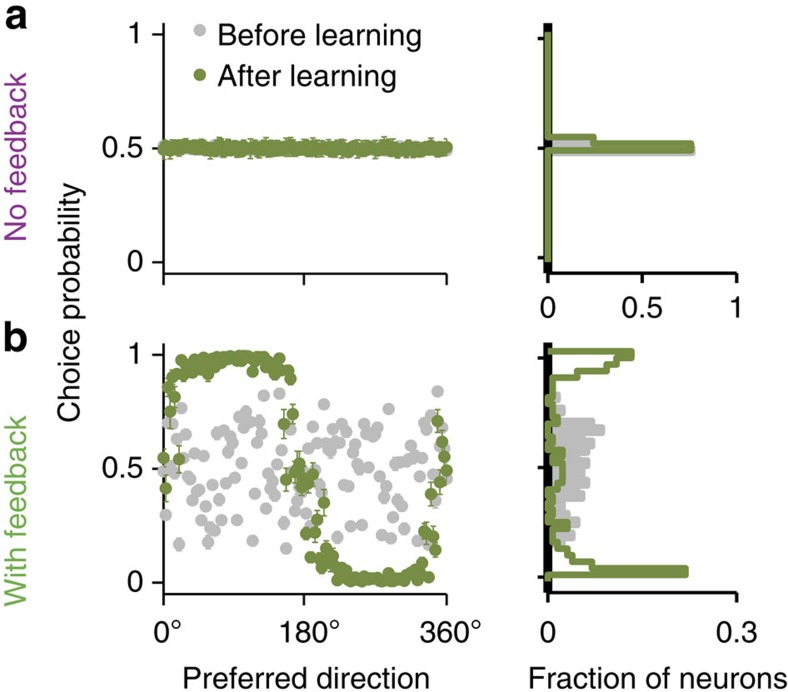
Association neurons in the network with feedback, but not in the network without feedback, exhibit choice-correlated fluctuations. (**a**) In the network without feedback, CP is close to 0.5 in all association neurons and does not change throughout learning. (**b**) In the network with feedback, CP is randomly scattered around 0.5 before learning (grey dots), but a bimodal profile of CP develops after a short period of learning (500 trials, green dots), such that CP>0.5 in neurons with preferred directions in category C1, and CP<0.5 in neurons with preferred directions in category C2. CP (*y* axis) is plotted for all association neurons labeled by their initial preferred direction (*x* axis) before (grey dots) and after (olive dots) learning. Histograms to the right show the corresponding CP distributions.

This general principle explains both the fast associative learning and slower behavioural improvements in our model. Since activities of decision neurons directly represent the model’s choices, the magnitude of their CP is large; hence, the synapses of decision neurons change rapidly towards increasing expected reward, underpinning fast associative learning. In the network with feedback, CP arises via feedback from the decision circuit, which produces multiplicative rate modulations in association neurons^30^,^31^ (Supplementary Fig. 2). Initially, CP is scattered around 0.5; however, when feedback connections become structured (~500 trials), neurons receiving stronger input from the C_1_ (C_2_) decision population fire at higher rates when *C*_1_ (*C*_2_) choices are made and exhibit CP>0.5 (CP<0.5, Fig. 6b). The magnitude of CP is smaller in association than in decision neurons; therefore, the tuning changes of association neurons and ensuing behavioural improvements happen more slowly than associative learning. In the network without feedback, CP≈0.5 in all association neurons and at all learning stages (Fig. 6a), because local noise in the decision circuit—required to attain realistic behavioural performance in the categorization task—diminishes the influence of association neurons' rate fluctuations on choices (see Supplementary Note 4 for details). Resulting unstructured synaptic changes lead to deterioration of tuning and behavioural performance. Regardless of which mechanism—feedforward or feedback—is more plausible for generating CP in real neurons, our results demonstrate the significance of CP for reward-dependent learning.

### Choice-correlated fluctuations shape neural tuning changes

**Figure 7:**
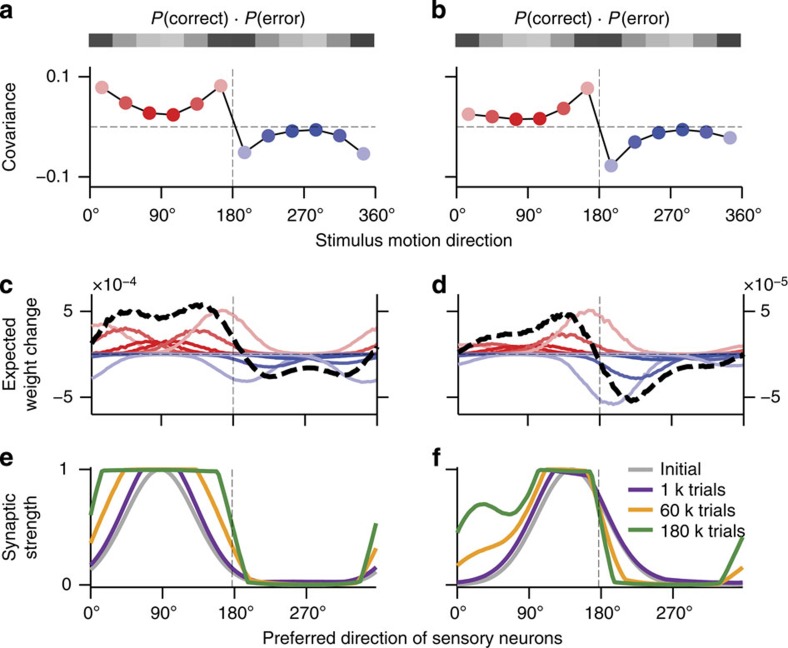
The covariance between reward and neural activity drives tuning changes in association neurons. (**a**,**b**) Covariance Cov[*R*, *r*_*i*_|*θ*] between the firing rate and reward (*y* axis) measured for each of the 12 motion direction stimuli (*x* axis) after 1,000 trials of learning. The covariance profile is shown for two association neurons with preferred directions near category centre (**a**) and near category boundary (**b**), the same sample neurons as in Fig. 3c. The covariance has opposing sign for stimuli in different categories (see equation 3). The magnitude of the covariance is proportional to the product of probability to make a correct response and the probability to make an error (grey-scale code bar above the graph), which is the largest for near-boundary stimuli. (**c**,**d**) The expected weight change for the synapses from all sensory neurons (*x* axis) to a single association neuron; **c**,**d** correspond to the association neurons shown in **a**,**b**, respectively. The weight changes expected for each stimulus (red lines—stimuli from category C1, blue lines—stimuli from C2, left *y* axis) add up to produce the total expected weight change (black dashed line, right *y* axis). (**e**,**f**) The weight changes from **c**,**d** accumulate over multiple trials. As a result, the synaptic profile from sensory neurons is gradually broadened for the association neuron tuned to the category centre, and shifted towards the category centre for the association neuron tuned near the category boundary. These changes of the synaptic profiles underlie neural tuning changes shown in Fig. 3c.

Over many trials, synaptic weight changes Δ*c*_*ij*_ between the association neuron *i* and sensory neurons *j*=1…*N* follow the same two trends as observed in tuning functions (Fig. 3c). For neurons tuned to category centres, the initial bell-shaped profile widens on both sides until it transforms into a step-like profile aligned with the category boundary (Fig. 7e); hence, the tuning curves broaden. For neurons tuned to directions near category boundaries, synapses are strengthened on one side and weakened on the other side of the initial bell-shaped profile (Fig. 7f); hence, the tuning curves shift towards the category centre. Using equation (2), the expected weight change for stimulus *θ* can be expressed as ‹Δ*c*_*ij*_|*θ*›=*q* Cov[*R*,*r*_*i*_*r*_*j*_|*θ*]≈*q* ‹*r*_*j*_|*θ*›Cov[*R*,*r*_*i*_|*θ*] (see Supplementary Note 2). The overall expected weight change is then the average of ‹Δ*c*_*ij*_|*θ*› across all stimuli. Thus, synaptic changes are determined by the covariance Cov[*R*,*r*_*i*_|*θ*] weighted by the rates of sensory neurons.

For neurons initially tuned to directions in category C1, CP>0.5 and the covariance Cov[*R*,*r*_*i*_|*θ*] is positive for stimuli *θ*εC1 and negative for *θ*εC2 (Fig. 7a,b), since the term (*R*_1,*θ*_−*R*_2,*θ*_) in equation (3) changes sign for *θ* in different categories. The covariance magnitude is proportional to the product of probabilities of the correct response and error, *P*_1,*θ*_(1−*P*_1,*θ*_), which is largest for near-boundary stimuli (*P*_1,*θ*_~0.5). When this covariance is combined with the firing rates of sensory neurons, the overall synaptic weight change is step-like for neurons tuned to category centres (Fig. 7c), and skewed towards the category centre for neurons tuned near category boundaries (Fig. 7d). For neurons initially tuned to directions in category C2, CP<0.5; hence, the covariance has just the opposite sign leading to the preference for category C2. Such tuning changes lead to behavioural improvements because the feedforward 
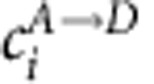
 and feedback 
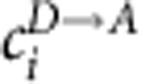
 connections become aligned through learning.

### Plastic top-down feedback induces task-specific correlations

In our model, category-tuning and neural fluctuations are simultaneously shaped through plasticity of feedforward and feedback connections to association neurons, giving rise to testable model predictions.

**Figure 8:**
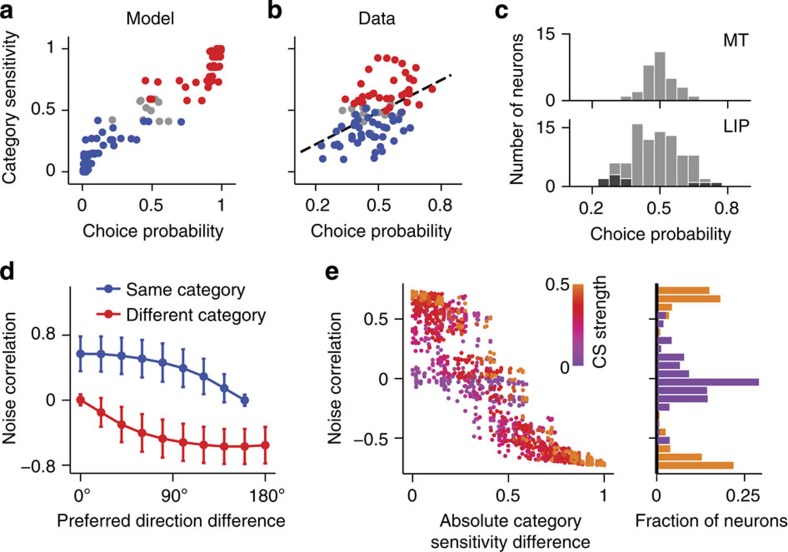
Model predicts interdependence between the CP, CS and noise correlations. (**a**) In the association neurons, a positive correlation between the CP and CS arises through learning. In the scatter plot, each dot represents a CP–CS pair for an association neuron (measured after 500 trials), colour-coded according to its category preference (C1—red, C2—blue, nonselective—grey). (**b**) A significant positive correlation between the CP and CS was found in LIP neurons recorded from behaving monkeys, similar to the model prediction in **a**. (**c**) Histograms of CP in MT and LIP populations. Black shading indicates neurons with individually significant CP. Overall magnitude of CP was significantly greater in LIP than in MT. (**d**,**e**) Plasticity of the feedback connections from decision neurons gives rise to task-specific noise correlations. (**d**) After learning, noise correlations decrease with the difference in preferred directions of two neurons, but are stronger for neurons preferring the same category, than for neurons preferring different categories. Error bars indicate s.d. across neurons. (**e**) Noise correlations are stronger in pairs of association neurons with more similar and individually larger CS. Scatter plot of noise correlation (*y* axis) versus the absolute difference between the category sensitivities (*x* axis). Each dot is coloured according to the average CS strength in the pair (purple-to-orange colour code corresponds to low-to-high CS strength). Histograms show the distributions of noise correlations for neural pairs with the high (>0.4) and low (<0.1) average CS strength (orange and purple, respectively).

First, our model predicts that association neurons with larger CP exhibit greater sensitivity of their tuning curve to the stimulus category (Fig. 8a). The latter is quantified by category sensitivity (CS), which is the accuracy with which an ideal observer could discriminate between stimuli from categories C1 and C2 given neuron’s firing rates on correct trials. A positive correlation between CP and CS arises because of reciprocal interaction of plasticity on the feedforward *c^S→A^* and feedback *c^D→A^* connections to association neurons. On one hand, plasticity of feedforward connections from sensory neurons leads to a greater increase in CS for neurons with larger CP (Fig. 5). On the other hand, plasticity of feedback connections from decision neurons generates a greater difference in top-down inputs from two decision populations, hence larger CP, for neurons with larger CS. The correlation between CP and CS is not an *a priori* given, because these measures quantify independent aspects of neuronal response. CS measures the difference in response to stimuli from different categories on correct trials, whereas CP measures the difference in response to the same stimulus on correct versus error trials. The correlation between CP and CS is abolished if the learned profile of feedback connections is randomized (Supplementary Fig. 6c).

We tested whether the predicted correlation between CP and CS exists in MT and LIP neurons. The overall magnitude of CP was significantly greater in LIP than in MT population (Wilcoxon rank-sum test comparing distributions of |CP−0.5|, *P*=0.0006, Fig. 8c). Ten LIP neurons (11.4%, *N*=88) and none of MT neurons (0%, *N*=31) showed individually significant CP (shuffle test with 1,000 shuffles and two-sample *t*-test, *P*<0.05, see Methods). In agreement with the model prediction, CP and CS were significantly correlated in the LIP (Fig. 8b, Pearson correlation, *r*=0.494, *N*=88, *P*=10^−6^), but not in MT population (*r*=−0.181, *N*=31, *P*=0.33, Supplementary Fig. 6d). We also repeated the analyses using CP computed relative to the preferred category of each neuron^17^,^29^ and obtained similar results (Supplementary Note 3 and Supplementary Fig. 6e–h). Although CP magnitude is slightly lower in LIP data than in the model, smaller CP magnitudes can be obtained in the model with weaker top-down connections (Supplementary Fig. 3). In addition, since recorded LIP neurons were sampled randomly, some of them might not be engaged in the categorization task and some were not visually responsive. This sampling heterogeneity may reduce the average effect size in the data and it is not incorporated in our model.

Second, our model predicts that interneuronal correlations depend on CS. In association neurons, correlations between their trial-to-trial rate fluctuations, termed noise correlations 
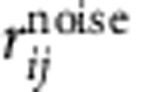
 (a Pearson correlation coefficient between rate fluctuations of neurons *i* and *j*), arise from shared recurrent and feedforward inputs. In the network without feedback, noise correlations simply decrease with the difference in neurons’ preferred directions reflecting the bell-shaped profile of their recurrent and feedforward connections (Supplementary Fig. 5b). In the network with feedback, association neurons with the same category preference also share top-down input from decision neurons, consequently noise correlations are stronger among neurons that contribute to the same category decision (Fig. 8d), similar to previous experimental reports^32^. Moreover, noise correlations are larger in neural pairs with smaller absolute difference in their category sensitivities (ΔCS_*ij*_=|CS_*i*_−CS_*j*_|): 
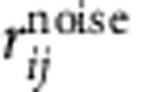
 is positive in pairs with similar CS (ΔCS_*ij*_~0) and negative in pairs with opposite category preference (ΔCS_*ij*_~1, Fig. 8e). In addition, the magnitude of noise correlations is larger in neural pairs with higher CS strength, defined as (|CS_*i*_−0.5|+|CS_*j*_−0.5|)/2 (Fig. 8e). Such structured noise correlations—that remained static throughout learning—were required in a feedforward model^10^,^29^ to capture the correlation between CP and task sensitivity observed in several experimental studies^7^,^8^,^29^,^33^. However, the *a priori* assumption that noise correlations depend on CS is not realistic, since categories are assigned arbitrarily. Alternatively, our model suggests that plasticity of feedback connections represents a common mechanism by which the structure of noise correlations, CP and CS all develop dynamically through learning.

## Discussion

Here we proposed a neural circuit mechanism for visual category learning. Our findings represent two major advances going beyond a model for categorization. First, we demonstrated that choice-correlated activity fluctuations, ubiquitous across cortical areas^7^,^8^,^9^,^17^,^34^, are critical for learning through reward-dependent Hebbian plasticity, which generally holds across different network architectures and behavioural tasks. Second, we showed how behavioural improvements, neuronal tuning changes, CP and noise correlations can be all simultaneously shaped by a common plasticity mechanism in a network incorporating top-down feedback. Several model predictions about ensuing interdependences between these measures were confirmed by the analysis of LIP recordings.

The reward-dependent Hebbian plasticity in our model belongs to the family of covariance-based learning rules^15^ using a stimulus-specific RPE signal, which is critical for successful learning^14^ (Supplementary Fig. 7). The idea to harness local fluctuations for reward-dependent learning has been first proposed for connectionist networks^35^, and later instantiated in networks of spiking neurons by exploiting either randomness of Poisson spiking^36^,^37^ or stochasticity of synaptic transmission^38^. Such plasticity rules can successfully learn precise spike patterns in networks of just a few neurons, but fail in larger networks and when behavioural outcomes are determined by population firing rates rather than by spike times of individual neurons^39^,^40^. The reason for their failure in these situations is precisely the lack of correlation between population-level choices and local activity fluctuations. To overcome this problem, plasticity rules have been employed incorporating behavioural choice explicitly as a multiplicative factor^10^,^41^,^42^. In contrast, our solution does not require any special plasticity rule, but instead utilizes network architecture where feedback from decision neurons generates choice-correlated variability.

Task-specific neural representations develop in many training paradigms across different cortical areas^43^,^44^,^45^,^46^,^47^,^48^,^49^,^50^,^51^,^52^,^53^,^54^,^55^. Our model demonstrates how such task-specific representations can emerge through reward-dependent plasticity. Although task-specific selectivity could arise through activity modulation via plastic feedback connections^56^, in our model, top-down modulation has a negligible effect on selectivity of association neurons (Supplementary Fig. 2), yet it is critical to guide learning of task-relevant features^57^.

Tuning changes of association neurons in our model allow for more accurate categorization of near-boundary stimuli than in the classical categorization model with fixed tuning^16^. In our model, tuning changes arise from plasticity of feedforward synapses from sensory (MT) to association (LIP) neurons; however, similar results are obtained if plasticity acts only on the recurrent synapses within the association circuit, or on both the feedforward and recurrent synapses (Supplementary Fig. 8). In our model, the initial direction tuning of association neurons sets the profile of choice-correlated fluctuations, which in turn governs tuning changes. However, initial tuning is not required for successful learning: a population of nonselective neurons carrying choice-correlated fluctuations develops categorical tuning just as well. In this case, neurons develop purely binary category selectivity with the category preference determined solely by their CP (Supplementary Fig. 9). Last, retraining on a categorization task with a new category boundary results in readjustment of neural tuning (Supplementary Fig. 10) similar to experimental observations^5^.

It has been speculated that category signals in LIP represent abstract perceptual decisions: category C1 versus C2 (ref. 58). In the motion categorization task, but not in classic motion discrimination work in LIP^7^, abstract decisions were dissociated from the actions signalling those decisions by using a two-interval match-to-category design, where the required motor response was unknown at the time of the first stimulus presentation. Moreover, receptive fields of LIP neurons in the motion discrimination task were aligned with the saccadic choice targets and not with the motion stimulus as in our case; hence, that design was better suited to examine response-related rather than perceptual signals in LIP. Accordingly, these data were interpreted using a feedforward model, where LIP neurons represent a decision-variable pooling activity of MT neurons with weights adjusted by a reinforcement learning rule^10^, and behavioural improvements were ascribed to selective strengthening of connections from the most sensitive sensory to decision neurons^8^,^10^. In contrast, we find that during motion categorization the representation of motion stimuli in LIP constitutes a mixture of directional and categorical tuning that facilitates discrimination of learned categories. Therefore, both mechanisms—that co-exist in our model—may be concurrently employed in the brain: refinements of sensory representations and of their readout by decision neurons.

In our model, mixed selectivity is robustly observed over a period from a few thousand to several hundred thousand trials, accompanied by increasing category tuning. Consistent with high CTI values reported previously in LIP^5^, we find that two factors contribute to the increasing population CTI: shift of preferred directions and emergence of mixed and pure category tuning. Some LIP neurons carried category selectivity throughout the delay period of the match-to-category task, which indicates that category encoding may not be a purely feedforward effect.

Our work demonstrates the significance of CP for reward-dependent learning regardless of its origin. The origin of CP has been recently debated, with accumulating evidence for top-down contributions^18^,^34^,^59^. Notably, CP signals we observe in LIP are distinct from signals related to reward, attention and upcoming movements^5^,^54^,^60^,^61^. Although origins of CP may differ between earlier sensory areas such as MT and more cognitive areas such as LIP, our model provides a common framework for understanding the impact of CP on plasticity of neuronal representation.

We proposed a novel model for how CP influences plasticity in LIP, although CP effects in sensory areas (for example, MT) have been modelled previously^18^,^29^,^62^. Our model demonstrates how a task-specific structure of CP, noise correlations and CS can arise dynamically through reward-dependent plasticity of top-down connections and predicts that neurons with larger CP develop larger CS. Thus, learning-induced tuning changes may be more pronounced in cortical areas that exhibit greater CP (Supplementary Fig. 3). Interestingly, both CP and CS were found to be significantly larger in LIP than in the prefrontal cortex^54^. Similarly, low CP of MT neurons might explain the absence of obvious tuning changes in this area through categorization training. Small but significant learning-related tuning changes have been observed in other sensory areas^4^ that also exhibit CP^63^. Therefore, our findings may generalize across sensory areas not limited to LIP.

## Methods

### Neural circuit model

*Network architecture*. The network model comprises three interconnected local circuits: sensory, association and decision. All three are strongly recurrent networks with dynamics governed by local excitation and feedback inhibition^19^,^20^,^64^. In simulations, we used a reduced mean-field model that has been shown to reproduce neural activity of a full spiking neural network^21^. The dynamics of each excitatory neural population is described by a single variable *s* representing the fraction of activated N-methyl-D-aspartate receptor conductance, governed by


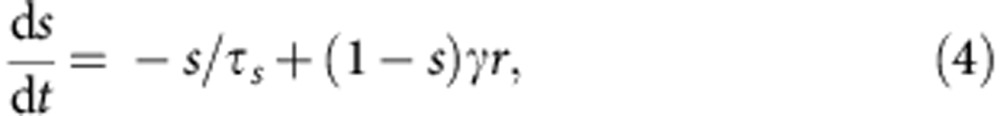


with *γ*=0.641 and *τ*_*s*_=60 ms. The firing rate *r* is a function of the total synaptic current *I* (refs 21, 65):


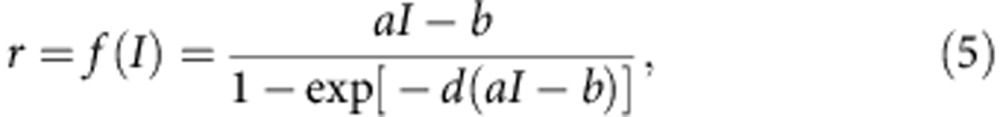


with *a*=270 Hz nA^−1^, *b*=108 Hz and *d*=0.154 s.

The total synaptic current *I* consists of recurrent and noisy components, *I*=*I*_*r*_+*I*_*n*_. Recurrent input to a neuron *i* in the population *A* originating from the population *B* reads:


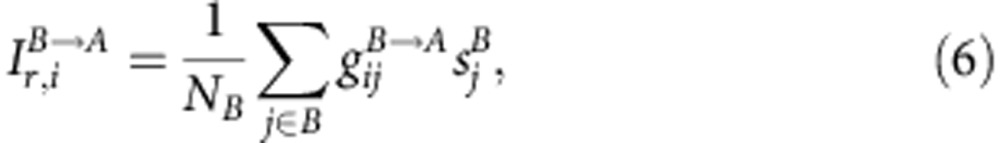


where 
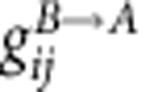
 is the synaptic coupling between the neuron *j* in the population *B* and the neuron *i* in the population *A*. The current is normalized by the number of presynaptic neurons *N*_*B*_. Noisy current replicates background synaptic inputs and obeys: 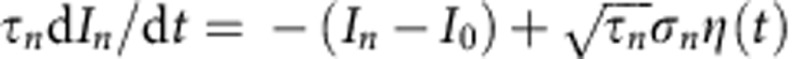, where *η*(*t*) is a white Gaussian noise, 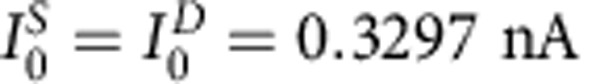, 
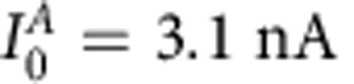
, *τ*_*n*_=2 ms and *σ*_*n*_=0.009 nA.

The sensory and association circuits were each simulated by 128 discrete units with equally spaced preferred directions from 0° to 360°. Within each circuit, the synaptic couplings *g*_*ij*_ between neurons with preferred directions *θ*_*i*_ and *θ*_*j*_ have a periodic Gaussian profile:


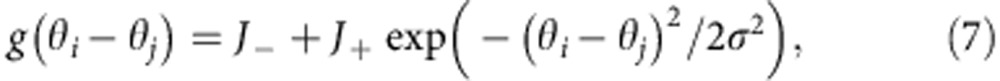


with *σ*=43.2°. Parameters *J*_−_ and *J*_+_ determine the amount of recurrent excitation and inhibition. In sensory and association networks, the recurrent inhibition is stronger than recurrent excitation, 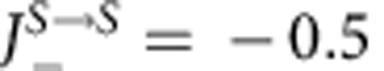, 
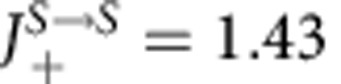
, 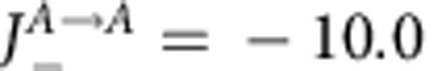 and 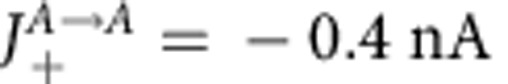. The particularly strong recurrent inhibition in the association circuit sets this module in the normalization regime^66^, where the total population activity remains approximately constant for different stimuli^19^.

The decision circuit consists of two populations (C_1_ and C_2_) representing categorical choice, which pool activity of the association neurons. When stimulated, activities of the C_1_ and C_2_ populations diverge according to winner-take-all dynamics. This behaviour is attained through global inhibition and structured recurrent excitation within the decision circuit^21^: 
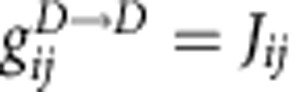
 with *J*_*C*1,*C*1_=*J*_*C*2,*C*2_=0.3725, nA, *J*_*C*1,*C*2_=*J*_*C*2,*C*1_=−0.1137, nA.

*Plastic synapses*. All synapses connecting three local circuits (from sensory to association, and between association and decision neurons) are plastic and excitatory. Synaptic strengths of plastic connections are expressed as *g*_*ij*_=*g*_max_*c*_*ij*_, where *g*_max_ is the maximal connection strength and *c*_*ij*_ is bounded between 0 and 1, and represents the fraction of potentiated synapses between neurons *i* and *j*. At the end of each trial, all *c*_*ij*_ are updated according to the Hebbain plasticity rule modulated by the RPE as specified in equation (1), where the learning rate *q*=0.00003, and *r*_pre_ and *r*_post_ are average firing rates during the stimulus period. The stimulus-specific predicted reward ‹*R*|*θ*› was estimated by a running trial average^14^: 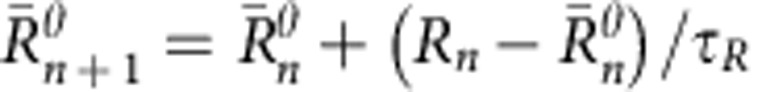, where *τ*_*R*_=5, and *n* enumerates trials with stimulus *θ*.

Plastic synapses between sensory and association neurons 
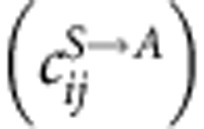
 were initialized with the periodic Gaussian profile as in equation (7) with 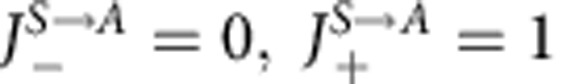. Plastic synapses between association and decision neurons (
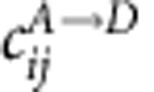
 and 
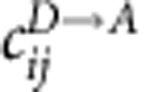
) were initialized randomly from a uniform distribution on [0.25, 0.75]. The maximal connection strengths of plastic synapses were 
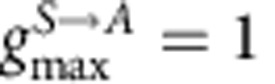
, 
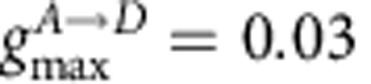
 and 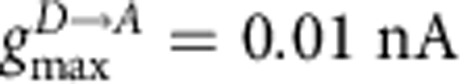.

### Simulation protocol and external inputs

Each simulation trial starts with a 200-ms pre-stimulus period (no external inputs), followed by a 1-s presentation of a motion direction stimulus and then by a 500-ms intertrial interval. When a motion direction stimulus *θ*_s_ is presented, neurons in the sensory network receive additional input current *I*_s_ that depends on the neuron’s preferred direction *θ*:


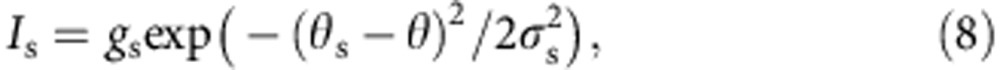


where *σ*_*s*_=43.2° and *g*_*s*_=0.1 nA. Neurons in the decision circuit receive a nonselective gating current of 0.01 nA during the stimulus period, which sets the circuit in the decision-making regime, and a brief −0.08 nA reset current during the first 300 ms of the intertrial interval, which represents the corollary discharge^67^ and resets activity to the spontaneous level.

The model’s response on each trial was determined by comparing firing rates of two decision populations with a 20-Hz threshold during the last 25-ms of the stimulus period. The response is considered invalid if both or neither population reach threshold, or either population reaches threshold before the stimulus onset. Across trials, choices of the decision network are stochastic and are characterized by a sigmoidal dependence of the probability of choice *C*_1_ on the difference Δ*I* in synaptic input currents to two competing populations^68^. Reward equals *R*=1 on valid correct trials, *R*=0 on valid incorrect trials and no plasticity is triggered on invalid trials.

Noise correlation 
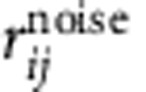
, CP and CS for the model neurons were estimated from 10,000 simulated trials with synapses ‘frozen’ (that is, no plasticity) at values attained after specified number of learning trials. Noise correlation 
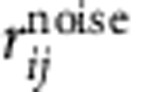
 was computed as the Pearson correlation coefficient between the firing rates of neurons *i* and *j* across all correct trials for the same stimulus, and then averaged across stimuli. CP and CS were computed as described in the Data analysis section, except for the CP estimation the model’s choice was known explicitly and did not have to be inferred.

Simulations were performed using a custom code written in Matlab implementing Heun integration with a time step of 1 ms. Code implementing the model is available upon request via email.

### Derivation of equation (3)

The covariance in equation (2), Cov[*R*,*N*|*θ*]=‹*RN*|*θ*›−‹*R*|*θ*›‹*N*|*θ*›, can be expressed in terms of expectations conditioned on the choice:


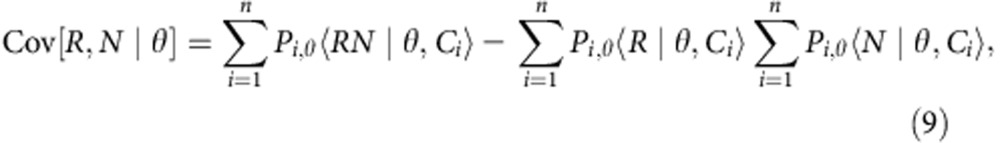


for a task with *n* possible choices *C*_*i*_, which are selected with probabilities *P*_*i*,*θ*_ for stimulus *θ*. In tasks where reward is delivered on the basis of behavioural response, the reward is independent of neural activity when conditioned on the choice; therefore,


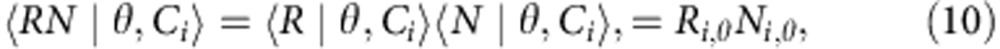


where *R*_*i*,*θ*_ and *N*_*i*,*θ*_ denote the conditional expectations of reward and neural activity, respectively, for choice *C*_*i*_ and stimulus *θ*. In these terms, equation (9) can be rewritten as


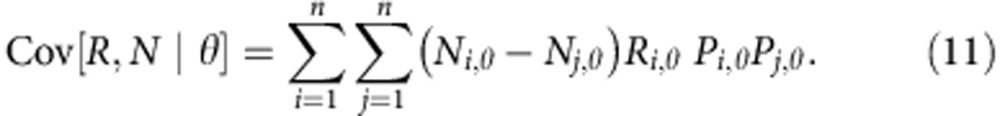


For tasks with only two possible choices, equation (11) simplifies to equation (3). In the categorization task reward is a deterministic function of choice (1 and 0 for correct and error choice, respectively); hence, the term (*R*_1,*θ*_−*R*_2,*θ*_) in equation (3) becomes +1 or −1 for stimuli *θ*εC1 or *θ*εC2, respectively.

### Toy-model neuron

We simulated a toy-model neuron (Fig. 5) to illustrate that CP drives synaptic changes independently of a particular network architecture and behavioural task. On each trial, a choice *C*_1_ or *C*_2_ was selected with probability 0.5. The firing rate of the toy-model neuron was then sampled from a Gaussian distribution with the mean *N*_*i*_ for choice *C*_*i*_ and variance 5 Hz. To generate different CP values, the following (*N*_1_,*N*_2_) pairs were used: (55, 50), (51, 50), (50, 50), (50, 51) and (50, 55) Hz. Synaptic changes were simulated with the plasticity rule in equation (1). For simplicity, the firing rate of neuron on the other synaptic side was assumed to be static through learning and set to 1. The mean firing rate and CP of the toy-model neuron were also assumed not to change through learning for simplicity. As in the circuit model, the predicted reward ‹*R*› was estimated by the running average with *τ*_*R*_=5, the learning rate was *q*=0.00003 and the synapse was initialized at 0.5.

### Behavioural task and neurophysiological recordings

All monkey data are from ref. 5, where experimental protocol and recording procedures were described in detail^69^. Two rhesus monkeys (*Macaca mulatta*, weighing about 14 kg) were trained to classify random-dot motion stimuli according to an arbitrary category boundary, which divided 360° of motion directions into two 180°-wide categories. Stimuli were circular patches (9° in diameter) of high-contrast square dots that moved with 100% motion coherence and at a speed of 12° s^−1^. Stimuli were always centred in the response field (RF) of the neuron under study. To dissociate categorical decisions from motor or premotor signals, the animals indicated category membership of the first stimulus (sample) by reporting (with a hand movement) whether it matched the category of the second stimulus (test). We focused on the categorization process of the sample stimulus and studied neural activity during the sample period (150–750 ms after stimulus onset, stimulus duration was 650 ms). To combine data from the two monkeys, all stimulus directions were rotated so that the category boundary was aligned with a 0°–180° axis.

The monkeys were implanted with a head post, scleral search coil and recording chamber. Recording chambers were implanted in accordance with coordinates (approximate centres at P3, L10) determined by magnetic resonance imaging, and allowed access to both the intraparietal sulcus (IPS) and the superior temporal sulcus by means of a dorsal approach. All surgical and experimental procedures followed the Harvard Medical School and National Institutes of Health guidelines. During LIP recordings, electrode penetrations sequentially encountered both the medial and lateral banks of the IPS. Most IPS neurons were tested with a memory-saccade task and a passive viewing flash-mapping task to generate detailed spatial maps of neuronal RFs. Neurons were considered to be in LIP if they showed spatially selective delay activity during the memory-saccade task or were located between such neurons in that electrode penetration. LIP neurons were not prescreened for direction selectivity. Area MT neurons were distinguished by direction-selective responses to moving spots and bars, and RF sizes that were roughly proportional to their eccentricity.

### Data analysis

*Tuning curve characterization*. The firing rates of MT and LIP neurons were transformed to standard *z*-scores. Tuning curves *r*(*θ*) were then constructed by computing average standardized firing rates in response to 12 motion direction stimuli *θ*. Tuning curves *r*(*θ*) of MT and LIP neurons, as well as those of association neurons in the circuit model were fitted by directional and categorical tuning profiles (least squares fit). The directional tuning profile was modelled by an exponential cosine function:


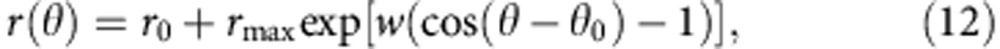


where *r*_0_ is the baseline firing rate, *r*_max_ is the peak amplitude, *w* is the tuning width parameter and *θ*_0_ is the preferred direction. First, we obtained the median tuning width *w* for each population from the unconstrained fit, and then refitted tuning curves with *w* constrained within the 10 percentile range around the median (93.4°–126.1° for MT and 101.4°–142.7° for LIP and association neurons) to avoid very broad low amplitude (that is, nearly flat) directional fits. The resulting median tuning width was 120.9° for LIP and 104.9° for MT neurons, similar to previous reports^6^. The categorical tuning profile was modelled by a step function:


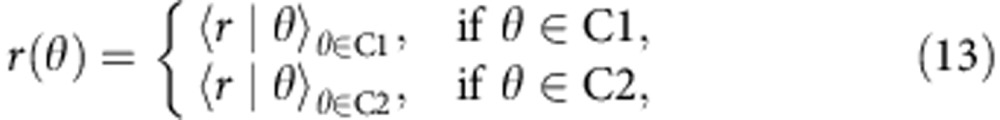


where 
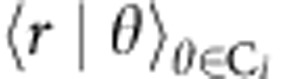
 is the average firing rate across stimuli in category C_*i*_. We repeated the analysis with more complex categorical tuning profiles (a periodic sigmoid function and a step-like function with a smoother firing rate change near category boundaries), and it did not change the conclusions of our study.

We then used a regularized GLM^26^ to determine the relative contribution of fitted directional and categorical tuning profiles to neural firing rates. Regularized GLM provides a principled way to assess the relative strength of direction and category tuning in each neuron, without overfitting and avoiding confounds because of correlation between direction and category-tuning profiles for neurons tuned to category centres. The regression algorithm solves the matrix equation *β*=(*X*^*T*^*X*+*λI*)^−1^*X*^*T*^*r*, where *X* is the matrix of three factors: fitted directional tuning profile, categorical tuning profile and a baseline, *r* is the vector of neuron’s firing rates across trials, *β* are the regression coefficients for each of the factors in *X* and *λ* is a ridge regression coefficient. The value of *λ* was chosen on the basis of a leave-one-trial-out cross-validation procedure, such that *λ* minimized the mean squared difference between predicted and actual firing rates^70^.

To determine whether the resulting *β* coefficients were significantly different from zero, we used a standard *t*-test to compare *β* against the distribution of shuffled *β* values, which was obtained by randomizing the trial order and then refitting the linear regression model (1,000 reshuffles). Each neuron was then classified as direction-tuned or category-tuned if the corresponding *β* was significantly different from zero (*P*<0.05), mixed direction- and category-tuned if both *β*’s were significantly different from zero and nonselective if neither *β* was significantly different from zero.

*CTI and CS*. The CTI measured the difference in firing rate (averaged across all trials for each direction) for each neuron between pairs of directions in different categories (a between-category difference) and the difference in activity between pairs of directions in the same category (a within-category difference). The CTI was defined as the difference between the within-category and between-category differences divided by their sum. Values of the index could vary from 1 (strong differences in activity to directions in the two categories) to −1 (large activity differences between directions in the same category, no difference between categories). A CTI value of 0 indicates the same difference in the firing rate between and within categories.

CS was estimated using a receiver-operating characteristic (ROC) analysis^17^ applied to the distributions of firing rates on correct trials with stimuli from categories C1 and C2. CS is the area under the ROC curve, which ranges between 0 and 1, and indicates the accuracy with which an ideal observer can assign category membership of a stimulus on the basis of the neuron’s trial-by-trial firing rate. Values of 1 and 0 correspond to strong preference for categories C1 and C2, respectively. Values of 0.5 indicate complete overlap of the firing rate distributions for the two categories, that is, no category selectivity.

*Estimation of CP in MT and LIP neurons*. CP was estimated on trials for which the test stimulus was far from (45° or 75°) the category boundary. The monkeys were proficient in categorizing such stimuli (97% correct when both sample and test were far from the boundary); therefore, we assumed that on these trials the test stimulus was categorized correctly and inferred the monkey’s decision about the sample category to be the same as the test category if the monkey responded match, and different category if the monkey responded nonmatch^54^. For each stimulus, CP was estimated using an ROC analysis applied to the distributions of firing rates on trials with different category decisions for the same stimulus (that is, correct versus error trials). CP is the area under the ROC curve that ranges between 0 and 1 and indicates the accuracy with which an ideal observer can predict the monkey’s category decision on a trial-by-trial basis given neuron’s firing rate. Values of 1 and 0 correspond to strong preference (higher firing rate) for *C*_1_ and *C*_2_ category decisions, respectively. Values of 0.5 indicate complete overlap of the firing rate distributions for two decisions. To reliably estimate CP, only stimuli with at least three trials for each category choice were included in the analysis, and only those neurons were included that had a valid CP estimate for at least one stimulus in each category, which resulted in 88 LIP and 31 MT neurons left for the analysis. The CP reported for each neuron was the average CP across all stimuli that passed the inclusion criteria. Significance of CP values for individual neurons was assessed with a shuffle test. To this end, choices of the monkey were randomly assigned to the firing rate data (separately for each stimulus), and then CP was recomputed (1,000 reshuffles). The actual CP was compared with the shuffled distribution with a two-sample *t*-test.

## Author contributions

T.A.E., W.C. and X.-J.W. designed research. T.A.E. and W.C. performed model simulations and analysed data. D.J.F. designed and performed experiments. T.A.E., W.C., D.J.F. and X.-J.W. discussed results and wrote the paper.

## Additional information

**How to cite this article:** Engel, T. A. *et al*. Choice-correlated activity fluctuations underlie learning of neuronal category representation. *Nat. Commun*. 6:6454 doi: 10.1038/ncomms7454 (2015).

## Supplementary Material

Supplementary Information Supplementary Figures 1-10, Supplementary Tables 1-2, Supplementary Notes 1-4 and Supplementary References
