# Supplementary material for: Choice-correlated activity fluctuations underlie learning of neuronal category representation
Source: Nat Commun. 2015 Mar 11;6:6454. doi: 10.1038/ncomms7454 (PMC4382677; doi:10.1038/ncomms7454)
Supplement: Supplementary Information — Supplementary Figures 1-10, Supplementary Tables 1-2, Supplementary Notes 1-4 and Supplementary References [file ncomms7454-s1.pdf]

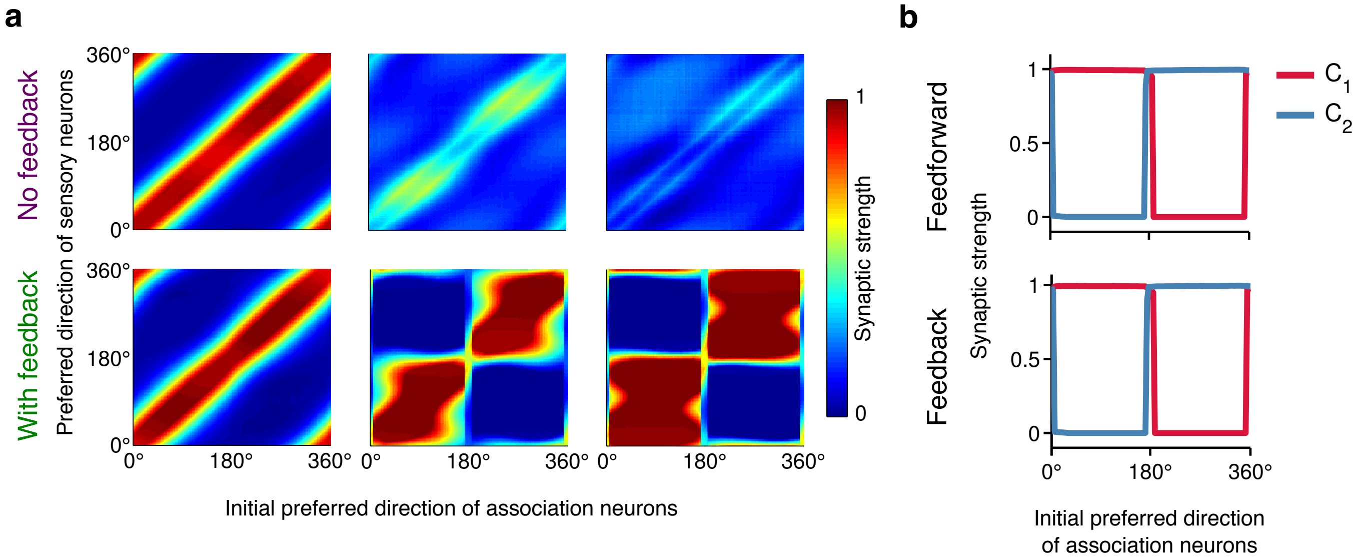

Supplementary Figure 1 | Changes of the synaptic connectivity profiles in the networks with and without feedback through the course of categorization training (**a**) Synaptic connectivity profiles from sensory to association neurons ($c^{S \to A}$) at the end of short (left column), intermediate (middle column) and long (right column) stages of training (as in Fig. 2) for the networks without (upper row) and with (lower row) feedback. x-axis, association neurons arranged and labeled by the preferred direction before learning; y-axis, sensory neurons labeled by the preferred direction; synaptic strength is color-coded. In the network without feedback, the initial bell-shaped periodic connectivity profile flattens in the course of learning, which leads to the loss of selectivity in the association neurons. In the network with feedback, a block-like connectivity profile develops through the course of learning, such that association neurons with initial preferred directions in category C1 receive nearly all their inputs from the sensory neurons with preferred directions in C1, and almost no inputs from sensory neurons with preferred directions in C2 (and vice versa for the association neurons with initial preferred directions in C2). The transformation of the connectivity profile from bell-shaped periodic towards block-like categorical underlies the emergence of category selectivity in the association neurons. (**b**) Profiles of the feedforward connections from the association to decision neurons ($c^{A \to D}$, left panel), and of the feedback connections from the decision to association neurons ($c^{D \to A}$, right panel) in the network with feedback after 160×10^3 trials of training (red and blue lines indicate connections to the C_1 and C_2 decision pools, respectively). The initially random profiles of the feedforward and feedback connections become nearly identical through the course of learning, since both sets of connections are subject to the same weight changes under the reward-dependent Hebbian plasticity.

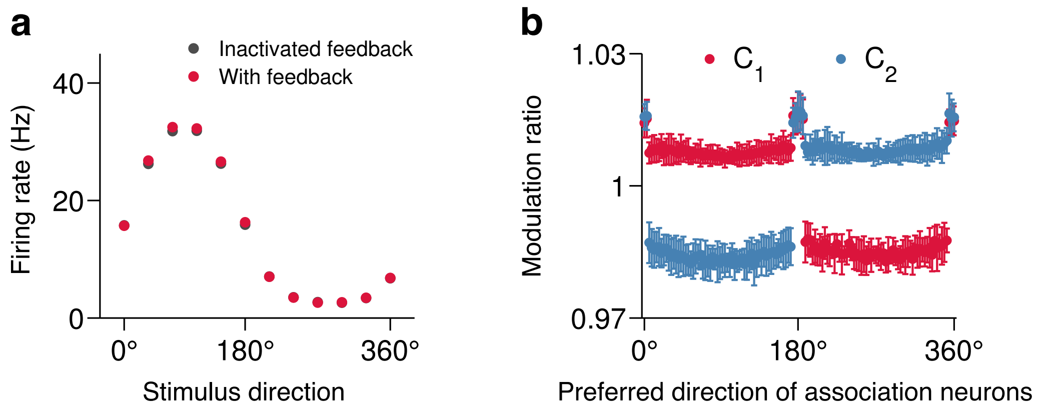

Supplementary Figure 2 | Effects of the feedback input from decision neurons on tuning of association neurons (**a**) Feedback input from the decision neurons does not affect the shape of tuning curves in association neurons. Tuning curve is shown for an example association neuron in the network with feedback measured on correct trials (i.e. for C_1 and C_2 choices for stimuli from categories C1 and C2, respectively) at the intermediate stage of learning (red dots). To isolate the contribution of the feedback input, the tuning curve of the same neuron was also measured in a modified network, where the feedback connections were inactivated ($c^{D\to A}$=0) while the learned strengths of all other connections were kept unchanged (grey dots). The firing rate modulation due to the feedback input is so small that its impact on the shape of the tuning curve is negligible. (**b**) Feedback input from decision neurons generates multiplicative scaling of tuning curves in association neurons. Modulation of activity by the feedback input can be quantified by the modulation ratio, which is the ratio of average firing rates in the networks with intact and inactivated decision feedback. For each association neuron, the modulation ratio was measured separately for each stimulus and separately for C_1 and C_2 category choices, on correct trials only. This implies, that for the C_1 and C_2 choices only stimuli from C1 and C2 categories, respectively, were included in the analysis. For each association neuron, the modulation ratio averaged across stimuli is shown for the C_1 (red dots) and C_2 (blue dots) choices, and the error bars indicate standard deviation across stimuli. For all neurons, the modulation ratio was nearly constant across different stimuli, indicative of a multiplicative scaling effect of the decision feedback on the tuning curves. For each category choice, activity is enhanced in neurons preferring the same category (modulation ratio > 1) and suppressed in neurons preferring the opposite category (modulation ratio < 1). Therefore activity modulation by the feedback input in our model is similar to the effects of top-down feature attention[1,2], with the difference that in our model modulation is binary discrete (“attention” to a category rather than to a continuous feature).

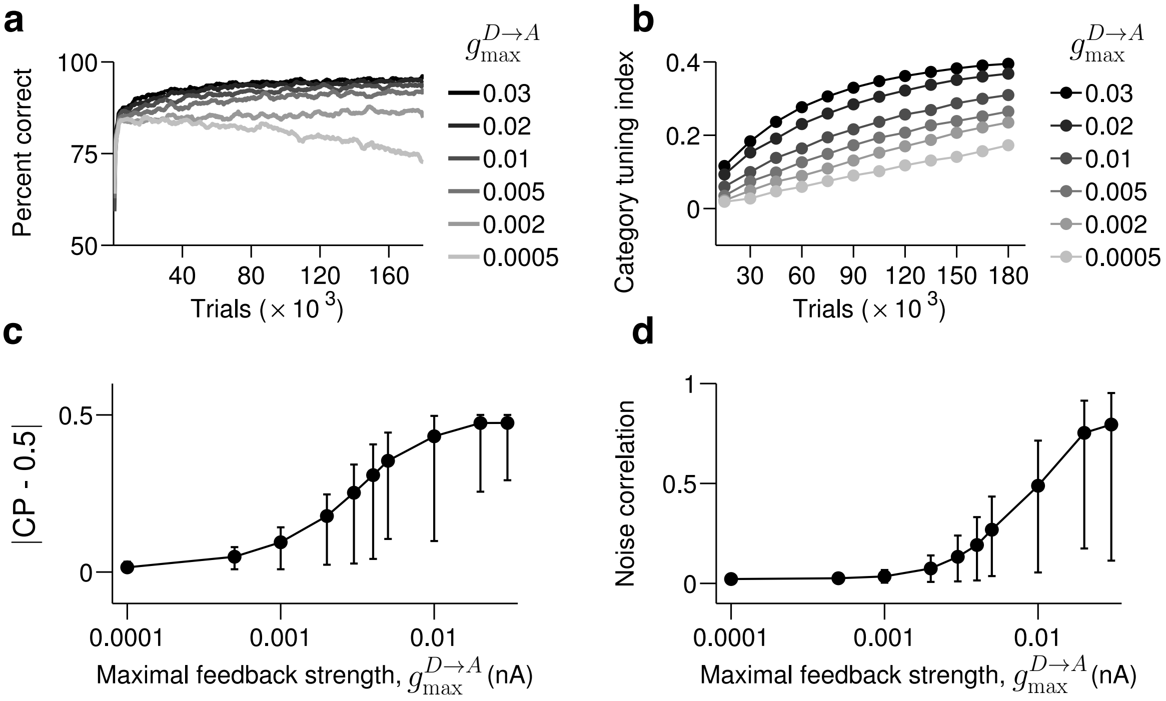

Supplementary Figure 3 | The strength of feedback connections from decision neurons controls the time course of learning, as well as the magnitude of category tuning, choice probability and noise correlations in the association neurons The same network model as in the main text, but with different maximal strength of the feedback connections $g_{\max}^{D\to A}$. Stronger feedback input results in greater average magnitude of CP (**c**) and greater average absolute value of noise correlations (**d**) in the association neurons. The expected synaptic weight changes are therefore greater with stronger top-down input (see Fig. 5), resulting in faster increase in the category tuning index of association neurons (**b**) and hence in faster behavioral improvements (overall precent correct responses, **a**). Error bars in **c** and **d** are $5\% - 95\%$ percentiles.

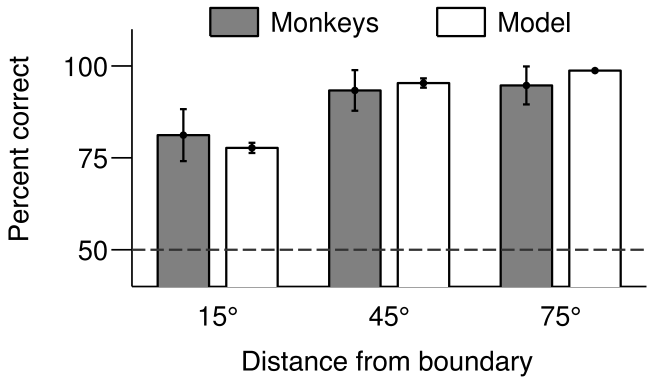

Supplementary Figure 4 | Behavioral performance on the motion categorization task was similar in the monkeys and neural circuit model Psychometric functions evaluated for monkeys during the recording sessions (grey bars) and for the neural circuit model at the end of intermediate training epoch (measured at 60×10^3 to 72×10^3 trials, white bars): percent correct responses for stimuli close to (15°) and farther from (45° and 75°) category boundary. The monkeys were trained for $\sim 40 - 80 \times 10^3$ trials prior to recordings, and recording sessions constituted $\sim 40 \times 10^3$ additional trials on average. Error bars indicate standard deviation across two animal subjects and across five independent network realizations, respectively.

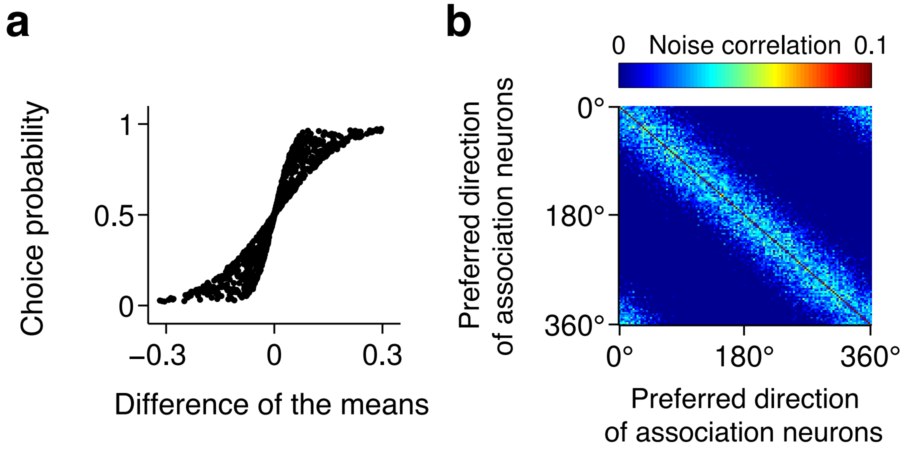

Supplementary Figure 5 | Choice probability and noise correlations in the neural circuit model (**a**) Choice probability is monotonically related to the difference between the means $N_{1,\theta} - N_{2,\theta}$ of two neural activity distributions, obtained on trials when the network picks different choices (C_1 and C_2) for the same stimulus θ. Scatter plot of CP (y-axis) versus $N_{1,\theta} - N_{2,\theta}$ (x-axis) for each neuron and each stimulus θ in the network with feedback prior to learning. (**b**) In the network without feedback, noise correlations r_{ij}^{noise} among association neurons decrease with the difference in their preferred directions. x-axis and y-axis, association neurons labeled by the preferred direction before learning; noise correlation is color-coded. The structure of noise correlations reflects the bell-shaped profile of the shared feedforward and recurrent inputs in the association circuit. The structure and magnitude of noise correlations can be tuned arbitrarily by injecting correlated noisy current I_n in the association circuit (see Methods). However, we did not attempt to fit noise correlations in the network without feedback and therefore used independent noise source $\eta(t)$ for each neuron.

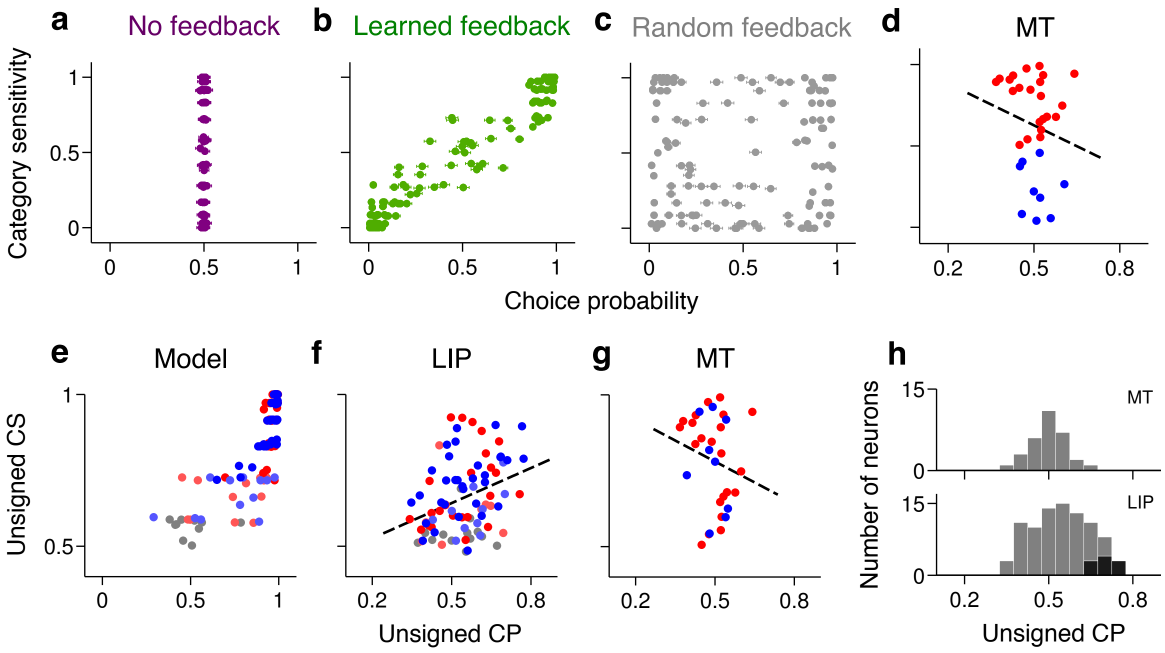

Supplementary Figure 6 | Model predicts a positive correlation between choice probability and category sensitivity after learning (**a-c**) Positive correlation between the choice probability (CP) and category sensitivity (CS) arises only in the network with learned profile of feedback connections from decision neurons. (**a**) In the network without feedback, CP ≈ 0.5 for all association neurons, hence no correlation between CP and CS exists. (**b**) CP and CS are positively correlated in the network with learned profile of feedback connections from decision neurons (same data as in Fig. 8a). The correlation between CP and CS arises because the learned profile of feedback connections is congruent with the profile of CS in association neurons. (**c**) The correlation between CP and CS is not given a priori, because these two measures quantify independent aspects of the neuronal response. CS measures the difference in response to stimuli from different categories on correct trials, whereas CP measures the difference in response to the same stimulus on correct vs. error trials. To demonstrate this independence, we measured CP and CS in the network, where the learned profile of feedback connections $c^{D\to A}$ was randomly shuffled between neurons. The CP and CS were not correlated in this network with random feedback. (**d**) Choice probability and category sensitivity were not correlated in our sample of MT neurons. In the scatter plot, each dot represents a CP-CS pair for an MT neuron, color-coded according to the neuron's category preference (C1 - red, C2 - blue). (**e-h**) Results were similar if we used unsigned CP and unsigned CS, i.e. CP and CS computed relative to the preferred category of each neuron[3]. Unsigned CP > 0.5 if neuron's preferred choice is the same as its preferred category, and unsigned CP < 0.5 if they are different. (**e**) Unsigned CP and unsigned CS are positively correlated in the network with learned profile of feedback connections from decision neurons due to the reciprocal interaction of plasticity on the feedforward $c^{S\to A}$ and feedback $c^{D\to A}$ connections to association neurons. (**f**) A significant positive correlation between the unsigned CP and unsigned CS was found in LIP neurons, similar to the model prediction in **e**. (**g**) Unsigned CP and unsigned CS were not correlated in our sample of MT neurons. (**h**) Histograms of unsigned CP in MT and LIP populations. Black shading indicates neurons with individually significant unsigned CP. See **Supplementary Note Supplementary Note 3** for statistical quantification of results in **f-h**.

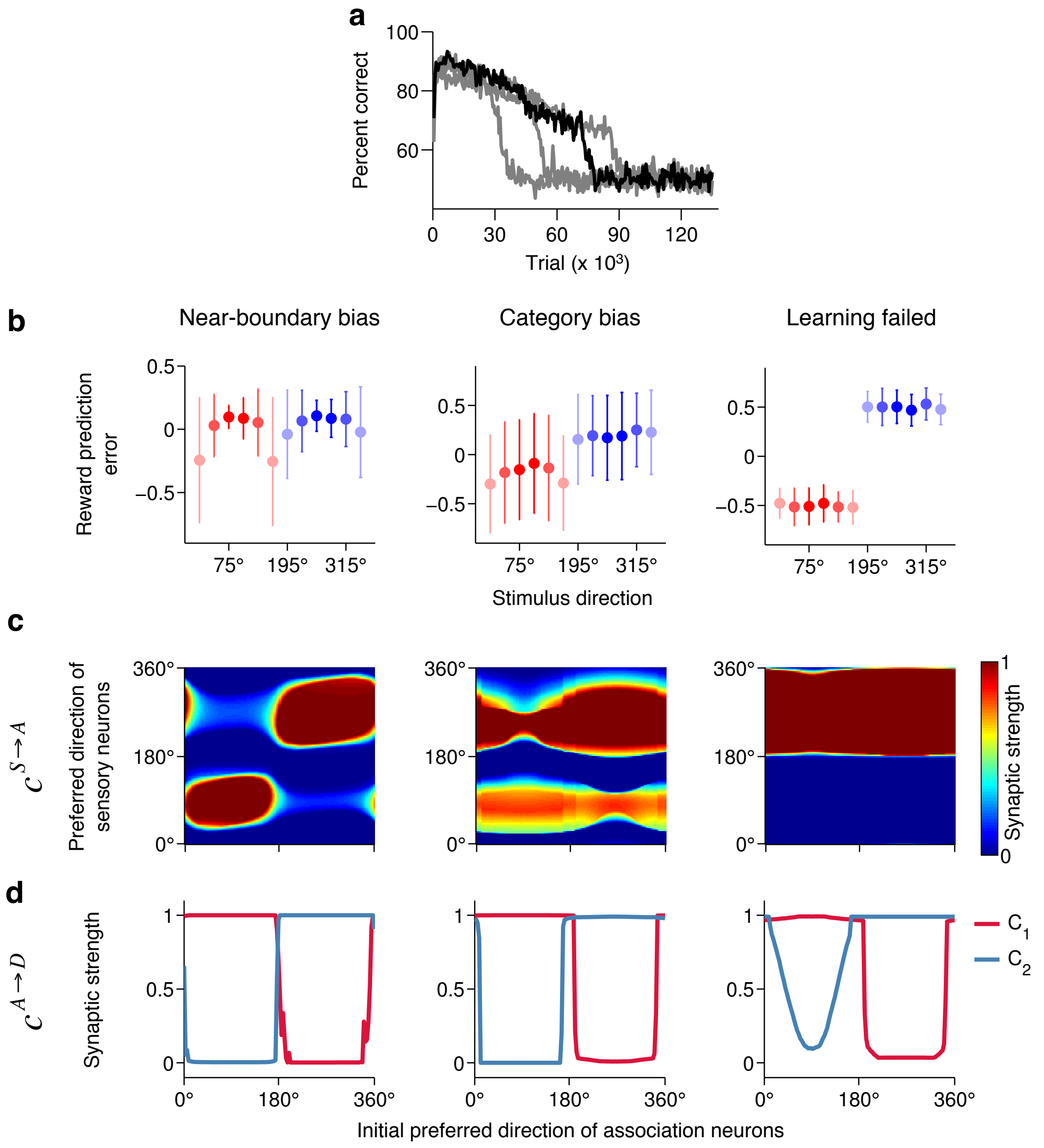

Supplementary Figure 7 | Stimulus-specific reward prediction error is critical for successful learning with Hebbian reward-dependent plasticity In our model, Hebbian synaptic plasticity is modulated by a stimulus-specific reward prediction error (RPE) $R - \langle R|\theta\rangle$, where $\langle R|\theta\rangle$ is the expected reward for stimulus θ. Learning critically depends on such stimulus-specific RPE signal and fails if non-selective (not stimulus-specific) RPE is used instead[4]. Indeed, consider a simplified scenario with only one stimulus per category ($\theta_1 \in$ C1 and $\theta_2 \in$ C2). Assume that for the current synaptic strengths, the probability of correct response (i.e. reward expectation) is not equal for the two stimuli, e.g., $\langle R|\theta_1\rangle > \langle R|\theta_2\rangle$. If the overall reward expectation $\langle R\rangle = [\langle R|\theta_1\rangle + \langle R|\theta_2\rangle]/2$ is used to compute RPE, then for θ_1 RPE is positive on average $\langle (R - \langle R\rangle)|\theta_1\rangle = \langle R|\theta_1\rangle - \langle R\rangle = [\langle R|\theta_1\rangle - \langle R|\theta_2\rangle]/2 > 0$, whereas for θ_2 RPE is negative on average $\langle (R - \langle R\rangle)|\theta_2\rangle = [\langle R|\theta_2\rangle - \langle R|\theta_1\rangle]/2 < 0$. Consequently, potentiation dominates for θ_1 leading to farther increase in the probability of correct response $\langle R|\theta_1\rangle$, and depression dominates for θ_2 leading to farther decrease in $\langle R|\theta_2\rangle$. Therefore small differences in the expected reward are amplified, until stimuli with slightly higher (lower) reward expectation almost

always (never) elicit correct response (i.e. $\langle R|\theta_1\rangle \approx 1$ and $\langle R|\theta_2\rangle \approx 0$). To demonstrate in simulations the importance of stimulus-specific RPE for successful learning, networks with feedback were trained on the motion categorization task with the modified plasticity rule, whereby the overall expected reward $\langle R\rangle$ (instead of $\langle R|\theta\rangle$) was used to compute RPE. In simulations $\langle R\rangle$ was estimated by a running trial average $\bar{R}_{n+1} = \bar{R}_n + (R_n - \bar{R}_n)/\tau_R$, where n is the trial number, and other parameters were the same as in the main text. (**a**) Percent correct responses as a function of the number of training trials for four network realizations. Similar to the main text result (Fig. 2), the performance rapidly rises to $> 80\%$ correct as networks learn associations between the motion directions and categories, but subsequently the performance gradually deteriorates and then sharply drops to the chance level at different time points during training for each network realization. (**b-d**) RPE and synaptic strengths for an example network (shown with a back line in **a**) at three time points during training: at the end of associative learning (6×10^3 trials, left panels), just before the performance drop (72×10^3 trials, middle panels), and after the performance drop (81×10^3 trials, right panels). (**b**) Average RPE for each motion stimulus θ (stimulus category is color-coded, error bars indicate standard deviation across trials). (**c,d**) Profiles of synaptic connections from sensory to association ($c^{S\to A}$, **c**) and from association to decision ($c^{A\to D}$, **d**) circuits. At the end of associative learning phase (left panels), the synapses $c^{A\to D}$ acquire a step-like profile aligned with the category boundary (**d**), but performance is less accurate for near-boundary stimuli than for stimuli near category centers (similar to the main text result, cf. Fig. 2d). Consequently, on average RPE is negative for near-boundary stimuli and positive for stimuli near category centers (**b**). This RPE bias leads to overwhelming depression of synapses $c^{S\to A}$ from sensory neurons with preferred directions near category boundaries, and to overwhelming potentiation of synapses $c^{S\to A}$ from sensory neurons with preferred directions near category centers (**c**), which results in gradual loss of selectivity for near-boundary stimuli in both categories and hence in gradual decline of performance. As soon as (by chance) the reward expectation becomes systematically larger for stimuli in one category (C2) than in the other (C1), this small discrepancy is amplified and leads to symmetry breaking (middle panels). The synapses $c^{S\to A}$ from sensory neurons tuned to directions in category C1 are depressed and in category C2 are potentiated (**c**), which amplifies the RPE bias (**b**) and results in performance drop. The performance ultimately drops to the chance level (right panels) and the network consistently chooses C_2 for all stimuli, when the synaptic profile $c^{S\to A}$ becomes nearly binary: connections are strong and vanishing from sensory neurons tuned to directions in categories C2 and C1, respectively, uniformly for all association neurons (**c**).

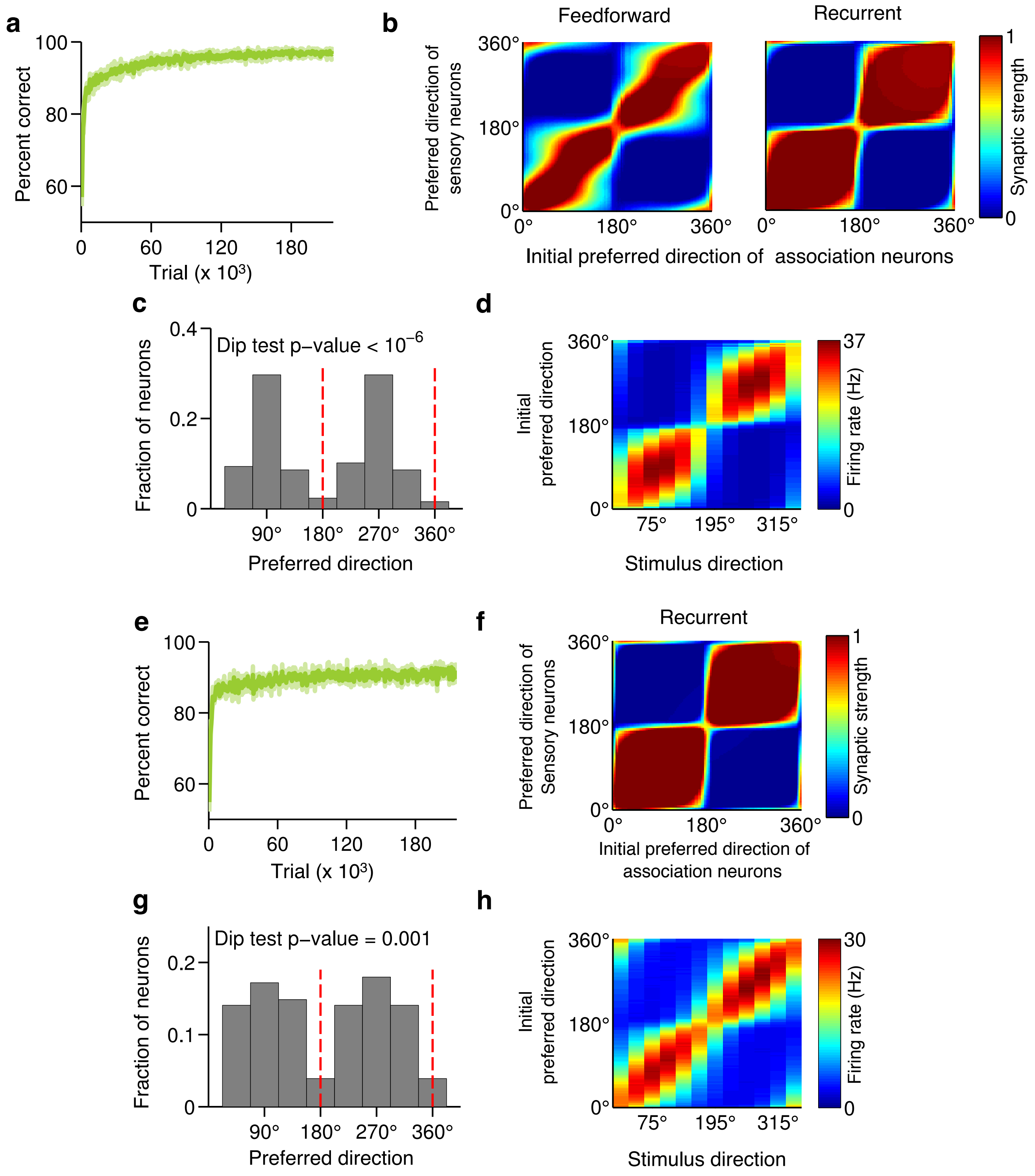

Supplementary Figure 8 | Categorization learning and tuning changes in the network with plastic recurrent connections in the association circuit Behavioral performance and tuning changes were similar in the network with plastic recurrent connections and in the network presented in the main text, where recurrent connections were not plastic. (**a-d**) Network with plasticity acting on the synaptic connections from sensory to association ($c^{S\to A}$, feedforward), from association to decision ($c^{A\to D}$, feedfor-

ward), from decision to association ($c^{D\to A}$, feedback) and between association neurons ($c^{A\to A}$, recurrent). (**e-h**) The same network as in **a-d**, but without plasticity on the feedforward connections from sensory to association neurons. In both network variants, only excitatory recurrent connections were plastic. The recurrent inhibition was global (equal weights between all neuron pairs) and not subject to plasticity. Other parameters are: $q = 0.00002, g^{D\to A} = 0.03$. Tuning curves and synaptic profiles were measured after 108×10^3 trials of categorization training. (**a,e**) Overall precent correct responses as a function of the number of learning trials. Similar to the results of the main text (Fig. 2), both network variants exhibit fast learning of associations between the motion directions and categories, followed by a gradual accuracy improvement especially for the near boundary stimuli. (**b,f**) Profiles of synaptic connections after categorization learning. In the presence of recurrent plasticity, the feedforward connections from sensory to association neurons develop a block-like categorical profile (**b**), similar to the network without recurrent plasticity (cf. Supplementary Fig. 1a). Through learning, the profile of recurrent connections between the association neurons also acquires the block-like categorical structure in both networks with recurrent plasticity (with and without plasticity on $c^{S\to A}$ synapses). (**c,g**) The distribution of preferred directions in direction-tuned association neurons was bimodal in both networks with recurrent plasticity, similar to the main text result (cf. Fig. 3d). (**d,h**) Tuning profiles of association neurons. x-axis, stimulus motion direction; y-axis, neurons arranged and labeled by their preferred direction before learning; firing rate is color-coded. Categorical tuning develops in both networks with recurrent plasticity similar to the main text result (cf. Fig. 3a), though the effect is slightly smaller in the network without plasticity on the feedforward connections $c^{S\to A}$.

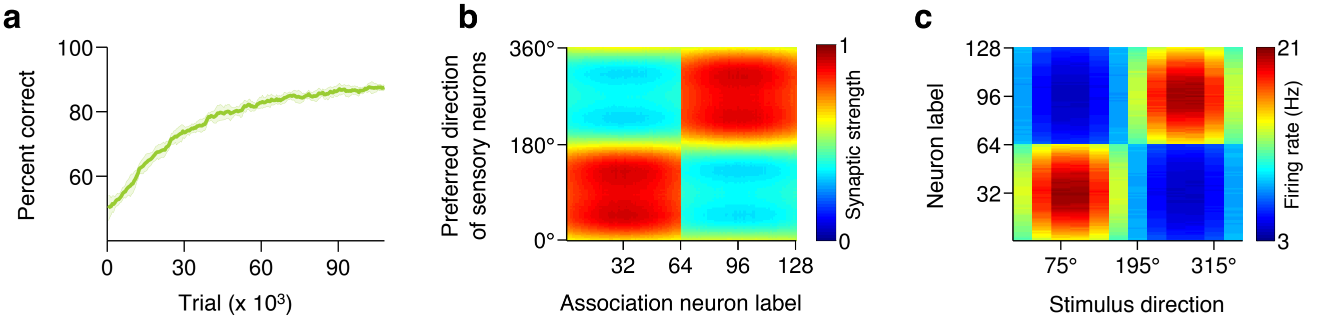

Supplementary Figure 9 | Categorization learning and emergence of tuning in the network with feedback, where association neurons are non-selective prior to learning Synaptic connections from sensory to association neurons $c^{S\to A}$ were initialized randomly from a uniform distribution on $[0.25, 0.75]$, therefore association neurons were not stimulus-selective prior to learning (flat tuning curve). The feedforward $c^{A\to D}$ and feedback $c^{D\to A}$ connections of decision neurons were not plastic and were set to the symmetric ($c^{A\to D} = c^{D\to A}$) step-like profile aligned with the category boundary (similar to the learned profile in Supplementary Fig. 1b), specifically $c^{A\to D}_{i,\mathrm{C}_1} = c^{A\to D}_{j,\mathrm{C}_2} = 1$ and $c^{A\to D}_{j,\mathrm{C}_1} = c^{A\to D}_{i,\mathrm{C}_2} = 0$ for $i = 1 \ldots 64$ and $j = 65 \ldots 128$. Other parameters are: $q = 0.00004, g^{D\to A} = 0.005$. This perfect correspondence between the feedforward and feedback connections ($c^{A\to D} = c^{D\to A}$) ensures that the profile of choice probability in the association neurons (determined by the feedback connections) is perfectly aligned with the contribution of each neuron to the decision (determined by the feedforward connections). Therefore tuning changes in association neurons lead to gradual behavioral improvements: neurons $i = 1 \ldots 64$, that project to the C_1 decision population and have CP > 0.5, develop preference for category C1, and neurons $j = 65 \ldots 128$, that project to the C_2 decision population and have CP < 0.5, develop preference for category C2. (**a**) Overall percent correct responses as a function of the number of performed trials. Note that network without initial tuning does not exhibit the fast associative learning at the short learning stage (cf. Fig. 2a), but shows gradual behavioral improvements throughout training caused by emerging tuning in association neurons. (**b**) Initially random profile of synaptic connections between the sensory and association neurons turns into a block-like categorical profile through learning. (**c**) Categorical tuning emerges in association neurons, which were initially untuned. x-axis, stimulus motion direction; y-axis, neurons; firing rate is color-coded. These results demonstrate that structured profile of choice probability matched to the profile of readout connections is sufficient for successful learning and for the emergence of category tuning in initially untuned association neurons.

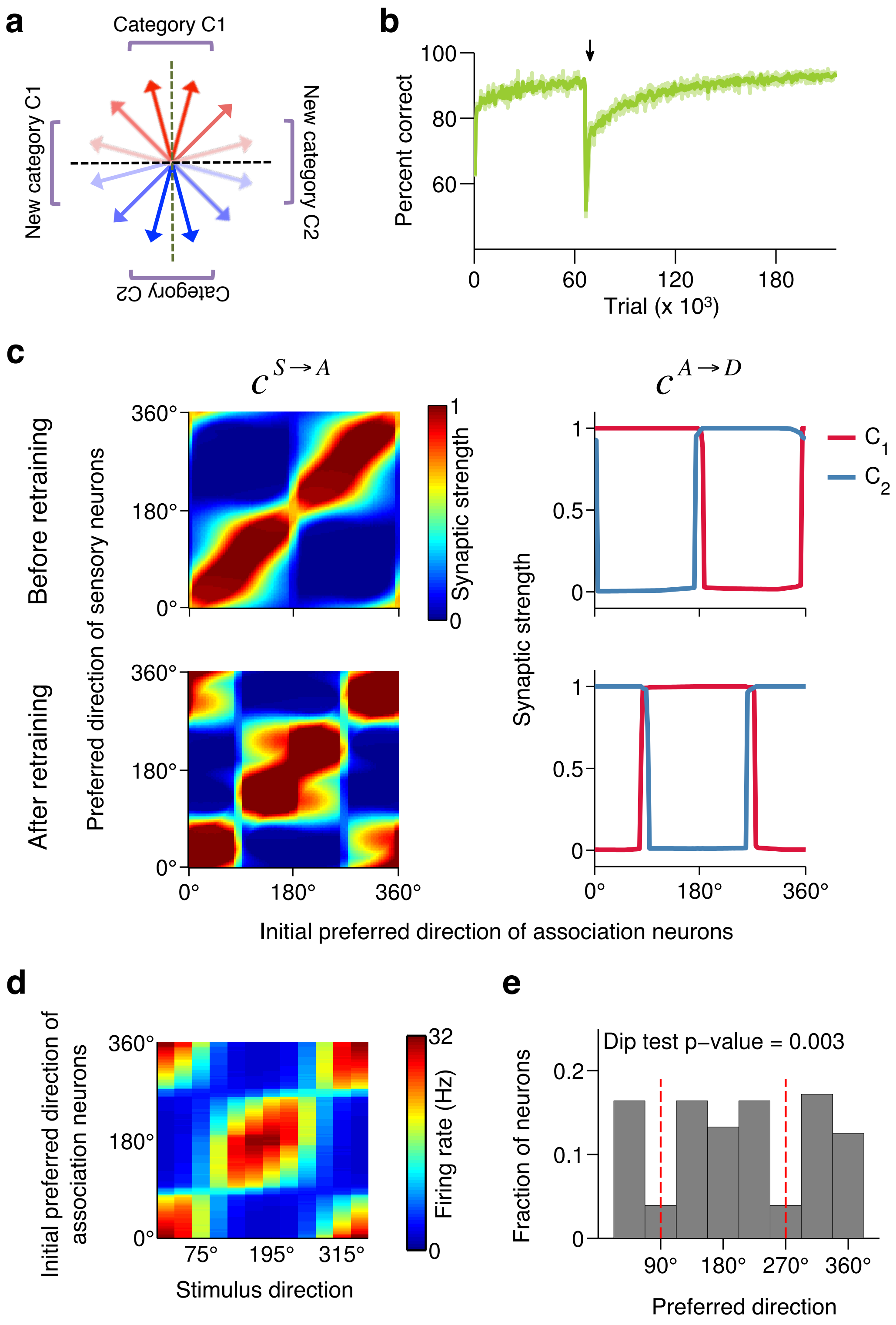

Supplementary Figure 10 | Category selectivity of association neurons follows retraining with a new category boundary (**a**) Network with feedback was first trained on the categorization task with the category boundary along $0° - 180°$ axis (black dashed line) for 65×10^3 trials, and then retrained with the new category boundary perpendicular to the original one (green dashed line, $90° - 270°$ axis) for

150×10^3 trials. (**b**) Percent correct responses as a function of the number of trials performed; black arrow indicates the trial when the new category boundary was introduced. Network's performance sharply drops to the chance level when the new boundary is introduced. Subsequently, the network exhibits the same two learning phases as observed during the initial categorization training (cf. Fig. 2): rapid behavioral improvements over few hundred trials corresponding to learning of associations between the motion stimuli and new categories, and subsequent slow performance improvements over several hundred thousand trials. (**c**) Profile of synaptic connections after the initial categorization training (upper panels), and after retraining with the new category boundary (lower panels), same format as in Supplementary Fig. 1. Synaptic connections from sensory to association ($c^{S\to A}$, left panels) and from association to decision ($c^{A\to D}$, right panels) neurons are shown. The profile of feedback connections from decision to association neurons ($c^{D\to A}$) is very similar to $c^{A\to D}$ (data not shown). Connections between the association and decision neurons ($c^{A\to D}$ and $c^{D\to A}$) acquire a step-like profile, which is aligned with the category boundary along $0° - 180°$ axis after initial training (upper right panel), and with the new category boundary along $90° - 270°$ axis after retraining (lower right panel). Corresponding changes in the profile of choice probability in the association circuit lead to rewiring of connections between the sensory and association neurons: after initial training $c^{S\to A}$ connections have a block-like structure aligned with the initial category boundary (upper left panel, cf. Supplementary Fig. 1a); after retraining $c^{S\to A}$ connections develop a similar block-like profile, but now aligned with the new category boundary (lower left panel). (**d**) These changes in the profile of synaptic connections $c^{S\to A}$ give rise to tuning changes in association neurons that follow the new category boundary. Association neurons initially tuned to directions in $90° - 270°$ range develop preference for the new category C1, while those initially tuned to directions in $0° - 90°$ and $270° - 360°$ range develop preference for the new category C2. x-axis, stimulus motion direction; y-axis, neurons arranged and labeled according to their preferred direction before learning; firing rate is color-coded. (**e**) After 65×10^3 trials of retraining with the new category boundary, the distribution of preferred directions in the association circuit is bimodal with more neurons tuned to the centers of new categories than to their boundaries (cf. Fig. 3d). Note that initial category boundaries at $0° - 180°$ correspond to the centers of new categories and vice versa.

Task Epoch (time window)	Direction	Category	Mixed	Non-selective
**Sample** (150−750 ms)	69.9	3.9	18.0	8.3
**Early Sample** (150−450 ms)	68.6	1.9	16.7	12.8
**Delay** (800−1700 ms)	47.4	5.1	21.2	26.3
**Early Delay** (650−1150 ms)	57.7	5.1	13.5	23.7
**Late Delay** (1150−1700 ms)	46.8	8.3	17.3	27.6

Supplementary Table 1 | Percentage of LIP neurons that exhibited directional tuning, category tuning, mixed tuning, or were not stimulus selective in different epochs of the delayed-match-to-category task[5] Time epoch is specified relative to the onset of the sample stimulus, and in all cases ends before the onset of the test stimulus. Proportion of category selective neurons slightly increases from the early sample to the late delay epoch, while proportion of direction tuned neurons decreases.

Task Epoch (time window)	Direction	Category	Mixed	Non-selective
**Sample** (150−750 ms)	66.0	7.1	17.3	9.6
**Delay** (800−1700 ms)	41.0	9.6	20.5	28.9

Supplementary Table 2 | Percentage of LIP neurons with directional, category or mixed tuning, or no stimulus selectivity Percentage are estimated in task epochs defined as in Supplementary Table 1, but using a less sharp category tuning function that allowed for a smoother firing rate variation near the category boundary: $r(\theta) = r_0 \pm r_1$ for stimuli farther from (45° and 75°) the category boundary, and $r(\theta) = r_0 \pm r_2$ for the near-boundary (15°) stimuli, where the $\pm$ sign is different for stimuli in different categories, and r_0, r_1, r_2 are fit parameters ($r_1 r_2 > 0$ and $|r_1| \geqslant |r_2|$). We used this smoother category tuning function to test for the possibility, that near-boundary stimuli may elicit weaker responses than stimuli near category centers even in category selective neurons, reflecting the fact that monkey's categorization accuracy was lower for near-boundary directions. First, an unconstrained fit was performed and the minimal $|r_1|$ and $|r_2|$ were determined among the neurons with significant category selectivity, and then the fit was repeated with parameters r_1 and r_2 constrained by these minimal values, to avoid low amplitude (nearly non-selective) fits. The estimated proportion of category tuned neurons is slightly higher than presented in the main text (Fig. 4b), but the overall trend is the same.

Supplementary Note 1

Quantifying category bias in the population-level representation

In the main text, MDS algorithm was used to visualize the population-level representation of motion directions in a two dimensional space. To quantify the degree of deviation from the uniform directional tuning in the population representation, we designed a distance-based measure called population category bias (PCB). PCB is similar to the category tuning index (CTI) described in Methods (Fig. 3b), but applies to the population activity rather than to activity of individual neurons.

To compute PCB, stimuli were represented as vectors in a high-dimensional space of neural firing rates, where each dimension corresponds to a neuron in the population (same as in the MDS framework). The euclidean distances (averaged across trials) between population representations are measured for direction pairs in the same category (a within category distance) and for direction pairs in different categories (a between category distance). The PCB was defined as the difference between the within category and between category distances divided by their sum. The PCB values could range from 1 (strong category bias, elongation along the axis perpendicular to the category boundary in the MDS plot, PCB $= 0.25$ in Fig. 3e, right) to -1 (elongation along the category boundary axis in the MDS plot). A PCB value of 0 indicates a uniform directional tuning in the population (no category bias, a perfectly circular configuration in the MDS plot, PCB $= 6.7 \cdot 10^{-4}$ in Fig. 3e, left).

To assess statistical significance of PCB values in MT and LIP populations, a bootstrap distribution of PCB was generated by computing PCB for 100 random subsets of trials (30% of available data). The mean PCB in the LIP population was 0.103, significantly larger than 0 (right-tail t-test, $p < 10^{-7}$), indicating that the population representation in LIP showed a significant category bias. The mean PCB in the MT population was -0.010, not significantly different from 0 (right-tail t-test, $p = 1.0$).

Supplementary Note 2

Plasticity rule computes covariance between reward and neural activity

On each trial, the synaptic weight change with the reward-modulated Hebbian plasticity rule (equation (1)) reads:

$$\Delta c_{ij} = q\,(R - \langle R|\theta\rangle)\, r_i\, r_j, \tag{1}$$

where r_i and r_j are the firing rates of pre- and postsynaptic neurons, and $\langle R|\theta\rangle$ is the reward expectation for stimulus θ given the current configuration of synapses in the network. The expected weight change reads[6]:

$$\langle \Delta c_{ij}|\theta\rangle = q\,\langle (R - \langle R|\theta\rangle)\, r_i r_j\rangle = q\,\mathrm{Cov}[R, r_i r_j|\theta]. \tag{2}$$

In the circuit model, the true reward expectation $\langle R|\theta\rangle$ on each trial is unknown and has to be estimated, for example by the running average $\bar{R}^{\theta}$ (see Methods). As a result of the discrepancy between $\langle R|\theta\rangle$ and $\bar{R}^{\theta}$, the expected weight change in the circuit model can deviate from the covariance $\mathrm{Cov}[R, r_i r_j|\theta]$:

$$\langle \Delta c_{ij}|\theta\rangle \approx q\,\langle (R - \bar{R}^{\theta})\, r_i r_j\rangle = q\,\mathrm{Cov}[R, r_i r_j|\theta] + q\,(\langle R|\theta\rangle - \langle \bar{R}^{\theta}\rangle)\langle r_i r_j\rangle. \tag{3}$$

The covariance term in equation (3) drives synaptic changes in the direction of increasing expected reward. The second term $q(\langle R|\theta\rangle - \langle \bar{R}^{\theta}\rangle)\langle r_i r_j\rangle$, however, depends only on the average reward and is therefore insensitive to covariation between reward and neural activity. This term reflects the mean behavior of the Hebbian component $\langle r_i r_j\rangle$ and introduces an unsupervised bias to the weight changes[4].

We estimated magnitudes of the covariance and of the Hebbian bias term for synapses between sensory and association neurons in our circuit model. The bias term is at least one order of magnitude smaller than the covariance term in a idealized stationary case, where the averages are computed over many trials ($\sim 10,000$) in a network with frozen synapses (no plasticity). In this stationary case, $\langle \bar{R}^{\theta}\rangle$ provides a very accurate estimate of the expected reward and their difference is very small. However, during actual learning, the synaptic strengths and reward probability are constantly changing, and the estimate $\langle \bar{R}^{\theta}\rangle$ is less accurate. As a result, during learning the Hebbian bias term has the same magnitude as the covariance term. In spite of that, the bias term does not undermine reward-dependent learning in our model, because its profile across neurons matches the profile of covariance. Namely, the covariance is positive for synapses between neurons with preferred directions in the same category, for which the Hebbian bias term is large and positive (due to large $\langle r_i r_j\rangle$ for neurons with similar preferred directions). Therefore Hebbian bias term drives synaptic changes in the same direction as the covariance for these synapses. The covariance is negative for synapses between neurons with preferred directions in different categories, but the Hebbian bias term is small for these synapses ($\langle r_i r_j\rangle$ is small for neurons with very different preferred directions) and has no impact on learning. In the network without initial tuning (Supplementary Fig. 9) the Hebbian bias term is also small compared to the covariance term due to smaller $\langle r_i r_j\rangle$ in untuned neurons.

Approximation for the covariance. The covariance $\mathrm{Cov}[R, r_i r_j|\theta]$ drives synaptic changes between the sensory and association neurons in the circuit model. To illustrate how these synapses acquire categorical profile through accumulation of weight changes Δc_{ij} over many trials (Fig. 7), we approximated the covariance

$$\mathrm{Cov}[R, r_i r_j|\theta] \approx \langle r_j|\theta\rangle \mathrm{Cov}[R, r_i|\theta]. \tag{4}$$

by pulling the firing rates of sensory neurons $\langle r_j|\theta\rangle$ out of the covariance (see main text). This is legitimate if firing rates of sensory neurons are not correlated with the reward R and firing rates of association neurons. To verify this in our circuit model, we expressed the covariance as

$$\mathrm{Cov}[R, r_i r_j|\theta] = \langle r_j|\theta\rangle \mathrm{Cov}[R, r_i|\theta] + \mathrm{Cov}[R\, r_i, r_j|\theta] - \langle R|\theta\rangle \mathrm{Cov}[r_i, r_j|\theta], \tag{5}$$

and estimated the order of magnitude for each term in the sum. The magnitudes were 10^{-2}, 10^{-5} and 10^{-5} for the first, second and third terms, respectively, which justifies dropping the last two terms in the approximation.

Supplementary Note 3

Correlation between unsigned choice probability and unsigned category sensitivity

Throughout the main text, we computed CP relative to the C_1 choice, i.e. by our convention CP > 0.5 if the firing rate is higher for C_1 choices, and CP < 0.5 if the firing rate is higher for C_2 choices. Therefore

the correlation between CP and category sensitivity reported in Fig. 8a-b reflects in part the alignment between neurons' preferred category and their preferred choice, i.e. the fact that CP tends to be greater than 0.5 for neurons preferring category C1 and smaller than 0.5 for neurons preferring category C2. Such alignment is not an a priori given. In our model, CP and CS become correlated after learning because the learned profile of feedback connections is congruent with the category preference of each neuron, but this correlation is abolished if the learned profile of feedback connections is randomized (Supplementary Fig. 6c). In general, preference for category C1 and C2 does not automatically imply CP > 0.5 and CP < 0.5, respectively. Therefore a significant correlation between CP and CS in LIP (Fig. 8b) could not be expected a priori. Indeed, CP and CS were uncorrelated in our sample of MT neurons (Supplementary Fig. 6d).

A slightly different definition of CP has been used in classic works by Britten and Shadlen[3,7], where CP of each neuron was evaluated relative to the choice corresponding to the preferred feature of its tuning curve. In our case, this means that CP is evaluated relative to the C_1 choice for neurons preferring category C1, and relative to the C_2 choice for neurons preferring category C2. Preferred category of each neuron was determined as the category that elicited higher mean response across all stimuli. We refer to CP evaluated this way as unsigned CP.

Unsigned CP is closer to 1 if the neuron's preferred choice is the same as its preferred category, and closer to 0 if they are different. Unsigned CP ≈ 0.5 if neuron's firing rate is not correlated with choices. Therefore, the population average of unsigned CP is different from 0.5 if neural activity is correlated with choices and neurons' preference for choice is aligned with their preference for category. In our example of the network model with randomized feedback connections, where the feedback connections are not aligned with neurons' category preference, the population-average unsigned CP ≈ 0.5, though firing rates of individual neurons are strongly correlated with choices (Supplementary Fig. 6c).

We evaluated the overall magnitude of unsigned CP in the MT and LIP populations (Supplementary Fig. 6h) and performed a permutation test to assess its statistical significance. To this end, monkey's choices were randomly assigned to the firing rate data (separately for each stimulus), unsigned CP was reestimated for each neuron and then population average was recomputed (1000 reshuffles). The population-average unsigned CP was then compared to the shuffle distribution of the population-average unsigned CP with a right-tailed two-sample t-test. Mean unsigned CP in LIP neurons was 0.55, significantly greater than chance based on the permutation test (right-tailed t-test, $N = 88, p < 10^{-4}$) and significantly greater than mean unsigned CP in MT (two-sample t-test, $p = 0.012$), the latter was 0.49, which was not significantly different from chance (right-tailed t-test, $N = 31, p = 0.93$). The mean of the shuffle distribution of the population-average unsigned CP was 0.500 in LIP (std $= 0.011$) and 0.499 in MT (std $= 0.014$), indicating that unsigned CP was not biased towards values greater than 0.5 (t-test, $p = 0.93$ and $p = 0.97$ in MT and LIP, respectively). We used a shuffle test (see Methods) to assess the number of MT and LIP neurons with significant unsigned CP. 10 LIP neurons ($11.4\%, N = 88$) and none of MT neurons ($0\%, N = 31$) showed individually significant unsigned CP (two sample t-test, 1000 shuffles, $p < 0.05$), all of them were the same neurons that also showed significant CP (Fig. 8c).

Similar to the results obtained in the main text, our model predicts a positive correlation between unsigned CP and unsigned CS (Supplementary Fig. 6f), which emerges from the reciprocal interaction of plasticity on the feedforward $c^{S \to A}$ and feedback $c^{D \to A}$ connections to association neurons

(as explained in the main text). Similar to the main text, the correlation between unsigned CP and unsigned CS was significant in LIP ($r = 0.293, N = 88, p = 0.006$, Supplementary Fig. 6f), but not in MT population ($r = -0.173, N = 31, p = 0.35$, Supplementary Fig. 6g).

Supplementary Note 4

Decision noise dominates choice probability in the feedforward model

In the model without feedback, CP ≈ 0.5 in all association neurons and at all learning stages (Fig. 6a). One could expect that rate fluctuations of association neurons should casually influence decisions, namely, that the C_i choice becomes more likely on trials when activity is higher than average in association neurons that have stronger connections to the C_i decision population. In the feedforward scenario, rate fluctuations would not influence choices, if all association neurons contributed with equal weights to both decision populations ($\Delta c_j^{A\to D} = c_{\mathrm{C}_1,j}^{A\to D} - c_{\mathrm{C}_2,j}^{A\to D} = 0$), or if rate fluctuations were independent among association neurons and averaged out for the population size we consider[3,8] (vanishing or unstructured noise correlations r_{ij}^{noise}). However, neither of these conditions applies in the network without feedback (see Supplementary Fig. 1b, 5b).

The reason for CP ≈ 0.5 in our model without feedback is that stochasticity of choices is dominated by the local noise in the decision circuit. Standard deviation of the feedforward input current

$$I_{C_i}^{A\to D} = g_{\text{max}}^{A\to D} \frac{1}{N_A} \sum_{j\in A} c_{C_i,j} s_j^D, \qquad (6)$$

from association to decision neurons (see equation (6)) equals $\sigma_{I^{A\to D}} = 0.00005$ nA (computed over the stimulus duration and averaged across stimuli, after 1000 learning trials), while the standard deviation of the local noisy current I_n in the decision circuit is $\sigma_{I_n} = 0.0061$ nA. Therefore variability of the feedforward input current $I^{A\to D}$ is very small compared to variability of the local decision noise.

High decision noise is needed to attain realistic behavioral performance in our categorization task, which employed strong stimuli (c.f. high-coherence motion in a classic motion discrimination task[7]). Strong stimuli elicit large difference in the mean input currents to two decision populations, strongly biasing the choice towards the population receiving larger mean input. This bias holds even for near-boundary stimuli, and even prior to learning due to slight imbalance in randomly initialized synapses. The feedforward fluctuations are not sufficient to overcome this strong bias, and without decision noise, the model would always pick the same choice for each stimulus (i.e. perform error-free, which was verified by setting $\sigma_n = 0$ in the decision circuit). Such deterministic behavior is not realistic, as monkeys make errors even for stimuli at the centers of categories (Supplementary Fig. 4). Moreover, stochastic choices (exploration) are required for learning with the reward-dependent Hebbian plasticity rule, since the covariance between reward and neural activity vanishes if $p_{i,\theta} = 0$ for one of the choices (see equation (3)).

The need for high decision noise is not an implementation feature of our decision circuit, but is a general consequence of using strong stimuli, which every model would have to face. To demonstrate this, we implemented a different readout scheme from the association neurons, the lin-

ear pooling model[3,8]. The pooling model computes a weighted sum of firing rates of association neurons $y = \sum_j \Delta c_j^{A\to D} r_j + \xi_{\text{pool}}$ and picks the choice C_1 if $y > 0$ and C_2 otherwise. The term ξ_{pool} represents the Gaussian decision noise with mean 0 and variance σ_{pool}^2. Without decision noise ($\sigma_{\text{pool}} = 0$), the choices of the pooling model in the categorization task were deterministic, just as in the circuit model. Computation of CP is not possible in this case. We determined the level of decision noise required to match the behavioral performance of the pooling model and monkeys (cf. Supplementary Fig. 4). This was achieved with $\sigma_{\text{pool}} = 8$ Hz, which was much larger than the variability of the pooled signal $\sigma_y = 0.6327$ Hz (averaged across stimuli). Such strong decision noise diminishes correlations between the firing rates of association neurons and choices resulting in CP ≈ 0.5 for all neurons (average unsigned CP $= 0.5031$).

To verify that the need for high decision noise does not result from some unrealistic features of the association circuit in our rate-based model (i.e. small population size, uniformity of tuning curves, unrealistic variability), we implemented a realistic population of sensory (MT-like) neurons following the method from ref.[9]. In brief, we simulated a population of 7200 motion-direction selective neurons with realistic heterogeneous tuning curves and response variability, and with noise correlations that decreased with the difference in neuronal preferred directions and motion sensitivities (see Methods in ref.[9]). The linear pooling model was then employed to form category decisions based on the responses of this large sensory population to motion stimuli, using a step-like categorical weights ($\Delta c = 1$ and $\Delta c = -1$ for neurons with preferred directions in categories C1 and C2, respectively). In ref.[9], the decision noise was found to be critical for modeling CP of MT neurons in a task, where subjects (and model) performed at sensory threshold discriminating low coherence motion stimuli. The match to experimental data was obtained with $\sigma_{\text{pool}} = 5$ Hz, which was approximately half the variability of the pooled signal σ_y. This level of decision noise is not sufficient to drive stochastic decisions in the categorization task with strong motion stimuli: the model performance was deterministic with $\sigma_{\text{pool}} = 5$ Hz. We found, that the monkeys' behavioral performance (Supplementary Fig. 4) can be matched if the decision noise is increased to $\sigma_{\text{pool}} = 80$ Hz, which largely abolished correlations between neural firing rates and choices (population-average unsigned CP $= 0.5096$).

In principle, stochastic choices for strong stimuli can also be achieved by increasing response variability of sensory neurons. We set $\sigma_{\text{pool}} = 0$, and increased the response variability of sensory neurons until the model's behavioral performance matched that of the monkeys. In this case, firing rates of sensory neurons were weakly correlated with choices (population-average unsigned CP $= 0.5668$). However, this was only possible to obtain with unrealistically high variability of sensory neurons, whereby the Fano factor (variance to mean ratio of spike count) ranged from 500 to 1000 in the population (a realistic value of Fano factor ~ 1 for cortical neurons).

In our circuit model, the ratio between the variance of the feedforward input current $I^{A\to D}$ and decision noise can be controlled by the parameter $g_{\text{max}}^{A\to D}$, the strength of feedforward connections (see equation (6)). To model the categorization task, we set $g_{\text{max}}^{A\to D}$ to be small (0.03 nA), to prevent the strong stimuli from generating very fast and deterministic decisions. In a task with weak stimuli, the impact of the feedforward input on choice can be enhanced by increasing $g_{\text{max}}^{A\to D}$. Our decision circuit model was previously shown to be able to capture monkeys' behavior at sensory threshold, in a task with low coherence stimuli[10] and in a fine discrimination task[11].

Supplementary References

1. Martinez-Trujillo, J. C. & Treue, S. Feature-based attention increases the selectivity of population responses in primate visual cortex. *Curr. Biol.* **14**, 744–751 (2004).

2. Ardid, S., Wang, X.-J. & Compte, A. An integrated microcircuit model of attentional processing in the neocortex. *J. Neurosci.* **27**, 8486–8495 (2007).

3. Shadlen, M. N., Britten, K. H., Newsome, W. T. & Movshon, J. A. A computational analysis of the relationship between neuronal and behavioral responses to visual motion. *J. Neurosci.* **16**, 1486–1510 (1996).

4. Fremaux, N., Sprekeler, H. & Gerstner, W. Functional requirements for reward-modulated spike-timing-dependent plasticity. *J. Neurosci.* **30**, 13326–13337 (2010).

5. Freedman, D. J. & Assad, J. A. Experience-dependent representation of visual categories in parietal cortex. *Nature* **443**, 85–88 (2006).

6. Loewenstein, Y. & Seung, H. S. Operant matching is a generic outcome of synaptic plasticity based on the covariance between reward and neural activity. *Proc. Natl. Acad. Sci. USA* **103**, 15224–15229 (2006).

7. Britten, K. H., Newsome, W. T., Shadlen, M. N., Celebrini, S. & Movshon, J. A. A relationship between behavioral choice and the visual responses of neurons in macaque MT. *Vis. Neurosci.* **13**, 87–100 (1996).

8. Haefner, R. M., Gerwinn, S., Macke, J. H. & Bethge, M. Inferring decoding strategies from choice probabilities in the presence of correlated variability. *Nat. Neurosci.* **16**, 235–242 (2013).

9. Law, C.-T. & Gold, J. I. Reinforcement learning can account for associative and perceptual learning on a visual-decision task. *Nat. Neurosci.* **12**, 655–663 (2009).

10. Wong, K.-F. & Wang, X.-J. A recurrent network mechanism of time integration in perceptual decisions. *J. Neurosci.* **26**, 1314–1328 (2006).

11. Engel, T. A. & Wang, X.-J. Same or different? A neural circuit mechanism of similarity-based pattern match decision making. *J. Neurosci.* **31**, 6982–6996 (2011).
